# Map of the Zintl AM_2_Pn_2_ Compounds: Influence of Chemistry on Stability and Electronic Structure

**DOI:** 10.1021/acs.chemmater.5c00353

**Published:** 2025-06-24

**Authors:** Andrew Pike, Zhenkun Yuan, Gideon Kassa, Muhammad Rubaiat Hasan, Smitakshi Goswami, Sita Dugu, Shaham Quadir, Andriy Zakutayev, Sage R. Bauers, Kirill Kovnir, Jifeng Liu, Geoffroy Hautier

**Affiliations:** 1 Thayer School of Engineering, 3728 Dartmouth College, Hanover, New Hampshire 03755, United States; 2 Department of Chemistry, 1177 Iowa State University, Ames, Iowa 50011, United States; 3 Department of Physics and Astronomy, 3728 Dartmouth College, Hanover, New Hampshire 03755, United States; 4 53405 National Renewable Energy Laboratory, Golden, Colorado 80401, United States; 5 Ames National Laboratory, U.S. Department of Energy, Ames, Iowa 50011, United States

## Abstract

The AM_2_Pn_2_ (A= Ca, Sr, Ba, Yb, Mg; M = Zn, Cd, Mg; and Pn = N, P, As, Sb, Bi) family of Zintl phases has been known as thermoelectric materials and has recently gained much attention for highly promising materials for solar absorbers in single-junction and tandem solar cells. In this paper, we will, from first principles, explore the entire family of AM_2_Pn_2_ compounds in terms of their ground-state structure, thermodynamic stability, and electronic structure. We also perform photoluminescence spectroscopy on bulk powder and thin film samples to verify our results, including the first measurements of the band gaps of SrCd_2_P_2_ and CaCd_2_P_2_. The AM_2_Pn_2_ compounds exhibit broad stability, are mostly isostructural to CaAl_2_Si_2_ (*P*3̅*m*1), and cover a wide range of band gaps from 0 to beyond 3 eV. This could make them useful for a variety of purposes, for which we propose several candidates, such as CaZn_2_N_2_ for tandem top cell solar absorbers and SrCd_2_Sb_2_ and CaZn_2_Sb_2_ for infrared detectors. By examining the band structures of the AM_2_Pn_2_, we find that Mg_3_Sb_2_ has the most promise as a thermoelectric material due to several off-Γ valence band pockets, which are unique to it among the compositions studied here.

## Graphical abstract

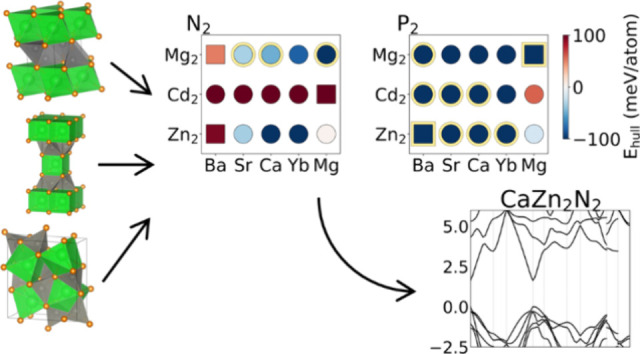

## Introduction

Zintl compounds make up a vast category of inorganic compounds that exhibit a combination of ionic and covalent bonding. Cations donate electrons, allowing a covalently bonded polyanion to form.^1–3^ Zintl compounds have a valence-precise composition, which allows for interchangeability of ions with like charges, forming new compounds similar to the parent. Examples of Zintl compound families include A_11_MPn_14_, A_21_M_4_Pn_18_, A_9_M_4_Pn_14_, and AM_2_Pn_2_, among many others, where the A cation is typically an alkaline-earth, alkali, or rare-earth metal, the M cation is a main group or transition metal, and Pn is a pnictogen.^4^ Zintl compounds are typically semiconductors because of the ionic nature of the interactions between cations and polyanions. Due to the narrow band gap of antimonides and bismuthides, they have mostly been investigated for their thermoelectric properties. The Zintl concept allows for straightforward tunability of the electronic structure, often coupled with complex unit cells, thus offering a unique platform to tune the electrical properties with low lattice thermal conductivity.

The AM_2_Pn_2_ compounds, sometimes called the “1–2–2” compounds, make up a compositionally diverse class of materials. We will restrict our investigation and discussion of the AM_2_Pn_2_ to A = Ca, Sr, Ba, Yb, and Mg; M = Zn, Cd, and Mg; and Pn = N, P, As, Sb, and Bi. Many of the original syntheses of the AM_2_Pn_2_ compounds were performed beginning in the late 1970s through the 1980s.^5–13^ From those syntheses, it was revealed that most of these compounds are isostructural, crystallizing in the CaAl_2_Si_2_-type structure (space group *P*3̅*m*1, No. 164) shown in Figure 1, and do not show any sensitivities to air or moisture exposure. In the past 20 years, the AM_2_Pn_2_ compounds crystallizing in the CaAl_2_Si_2_-type structure with small to intermediate band gaps have attracted attention for potential applications in heat-to-electricity conversion as thermoelectric materials, such as in systems based on YbZn_2_Sb_2_ ^14–16^ and Mg_3_Sb_2_.^17–20^ Very recently, it was discovered via high-throughput computational screening that BaCd_2_P_2_ and CaZn_2_P_2_ are promising thin film solar absorbers with high optical absorption in the visible light range and favorable defect properties, meaning the absence of low formation energy and deep intrinsic defects, allowing for long carrier lifetimes.^21,22^ Other AM_2_Pn_2_ are now being investigated for energy harvesting applications.^23–25^ Driven by these successes, we are motivated to search the entire family of AM_2_Pn_2_ compounds for other promising material candidates for thermoelectric devices, photovoltaic solar cells, and infrared detectors.

**Figure 1:**
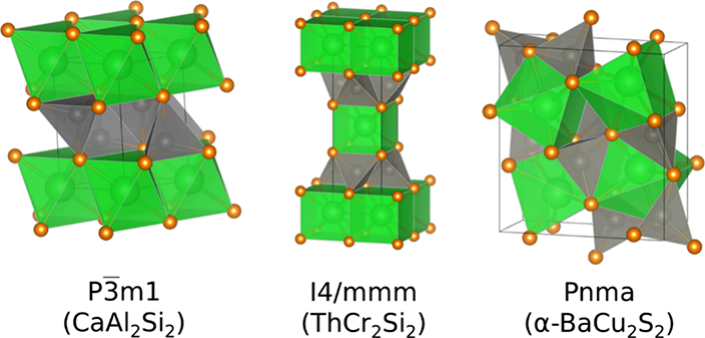
Three crystal structure prototypes for the AM_2_Pn_2_ compounds used in the ground-state structure search. Here the A element is green, M is gray, and Pn is orange. Drawings produced with VESTA.^47^

Here, we have systematically studied the phase stability and electronic band structure of the AM_2_Pn_2_ compounds in a wide range of compositions, using first-principles calculations, finding that the majority of compositions studied here are stable and isostructural and that these possess a wide range of band gaps. The band gap generally increases as Pn mass decreases; while bismuthides are mostly metallic, certain nitrides have band gaps greater than 3 eV. We compare the results to experimental literature and complement them with new experimental results. Our work can be used to understand well-established AM_2_Pn_2_ compounds, suggest new AM_2_Pn_2_ compounds to synthesize, and suggest specific applications for certain chemistries.

## Methodology

Our first-principles calculations were performed using the projector augmented-wave (PAW) pseudopotential method as implemented in the VASP code.^26–30^ All the calculations were automated using the Atomate code, including the generation of VASP input files.^31^ To determine the equilibrium crystal structure for each composition, we considered each AM_2_Pn_2_ adopting three different structures, shown in Figure 1, and these were first relaxed with full degrees of freedom using the PBE functional, which became the starting point for further calculations.^32^ For the PBE structure relaxations, an energy cutoff of 520 eV was used for the plane wave basis set. The convergence criteria for the self-consistent field (SCF) loop were set to 5 × 10^–5^ eV/atom, and the reciprocal **k**-point density was set to 64 Å^–3^. The tetrahedral method with Blöchl corrections without smearing was used to set the partial occupancies for each orbital. These parameters were chosen to be consistent with the Material Project database at the time of publication of this manuscript.^33^ Table S5 lists the pseudopotential versions used. For Yb, the f-electrons were considered frozen in the core. We did not include a Hubbard U parameter in any of the calculations in this study.

Next, we performed a further full geometry optimization, using the restored and regularized strongly constrained and appropriately normed (r^2^SCAN) functional.^34,35^ For the r^2^SCAN calculations, an increased plane wave energy cutoff of 680 eV was used, the smallest distance allowed between **k**-points was 0.22 Å^–3^, the convergence criteria for the energy of the SCF loop was 10^–5^ eV, the ionic loop was considered converged when the forces was less than 0.02 eV/ Å, the second order Methfessel-Paxton method of smearing was used with a width of 0.2 eV, and the PBE_54 set of pseudopotentials was used. The ground-state structure was determined by comparing the calculated total energies of the different polymorphs tested. Since we fully optimized the lattice parameters with r^2^SCAN using the PBE-relaxed structure merely as a starting point, the thermodynamic conclusions are unaffected by errors in the lattice parameters from the PBE relaxation.

To determine the 0K phase stability, competition with other elemental, binary, and ternary phases must be considered. To do this using the r^2^SCAN functional, the decomposition products of each AM_2_Pn_2_ were queried from the Materials Project (MP, which uses primarily PBE-GGA for its stability calculations) and relaxed in r^2^SCAN to be considered in the following reaction:

$$\mathrm{MP}\;\mathrm{Decomposition}\to {\mathrm{AM}}_{2}{\mathrm{Pn}}_{2}$$ 1

This prevents the calculation of the entire phase diagram, reducing computational cost, but gives a realistic reference for the phase stability rather than a formation energy calculated relative to the energy of the pure elemental phases. From the calculated total energies, the decomposition energy can be calculated to allow for assessment of the stability of the AM_2_Pn_2_.

$$E_{\mathrm{hull}}=E_{{\mathrm{AM}}_{2}{\mathrm{Pn}}_{2}}-\Sigma n\times E_{\mathrm{products}}$$ 2

where *n* is the reaction coefficient and *E*_products_ and *E*_AM_2_Pn_2__ are the total energies of the decomposition products and the AM_2_Pn_2_ compound of interest, respectively. Equation 1 represents the chemical reaction studied in order to calculate the *E*_hull_ in Equation 2. Here, negative *E*_hull_ indicates that a phase is thermodynamically stable. Taking BaCd_2_As_2_ as an example, the Materials Project predicts that it lies in a region of the Ba–Cd–As phase diagram that is bounded by Ba_2_Cd_2_As_3_, Cd_3_As_2_, and Cd, so for its decomposition, we consider the reaction:

$$0.5{\mathrm{Ba}}_{2}{\mathrm{Cd}}_{2}{\mathrm{As}}_{3}+0.25{\mathrm{Cd}}_{3}{\mathrm{As}}_{2}+0.25\mathrm{Cd}\to {\mathrm{BaCd}}_{2}{\mathrm{As}}_{2}$$ 3

So, its *E*_hull_ would be

$$E_{\mathrm{hull},{\mathrm{BaCd}}_{2}{\mathrm{As}}_{2}}=E_{{\mathrm{BaCd}}_{2}{\mathrm{As}}_{2}}-0.5E_{{\mathrm{Ba}}_{2}{\mathrm{Cd}}_{2}{\mathrm{As}}_{3}}-0.25E_{{\mathrm{Cd}}_{3}{\mathrm{As}}_{2}}-0.25E_{\mathrm{Cd}}$$ 4

For clarity, we have used the term *E*_hull_ here since it is well understood from other similar investigations, but we note that for the sake of this paper, we have defined it slightly differently than the common definition. Usually, the *E*_hull_ is calculated from the decomposition of the compound being investigated based on its full phase diagram, but we did not explicitly compute the entire phase diagram to get the full hull. For computational efficiency, we have calculated with r^2^SCAN only a limited set of compounds for each chemical system, relying on the Materials Project prediction of the decomposition, which is done at the PBE level of theory. In practice, this should be identical to the *E*_*hull*_ calculated from r^2^SCAN, except for the case where the set of compounds on the hull changes when calculated in r^2^SCAN compared to those reported on the Materials Project.

To determine the electronic band structure, noncollinear hybrid functional calculations using the parametrization of Heyd, Scuseria, and Ernzerhof (HSE06) including spin–orbit coupling (SOC) were performed based on the PBE-relaxed structures using a plane wave energy cutoff of 520 eV, the PBE_54 set of pseudopotentials, and the HSE mixing parameter (α) was set to 0.25.^36,37^ For the HSE band structure calculations, we used a self-consistent k-point grid with a reciprocal density of 50 Å^–3^ and virtual (i.e., zero-weight) k-points along a high symmetry path with a density of 10/unit cell along each segment in the path through the Brillouin zone. Computing HSE band structures on PBE-relaxed structures saves a significant amount of computational time compared to HSE band structure calculations on structures also relaxed from HSE. In this way, the calculations balance accuracy and computational cost. We have computed the carrier effective masses without including SOC. We do not expect significant differences between an effective mass calculated from an HSE and HSE + SOC band structure, since the difference in functional should not significantly affect the shape of the bands near the band gap. The conductivity effective mass tensor was computed by using Boltztrap2. This conductivity effective mass effectively takes into account the effects of band nonparabolicity and multiple bands’ contributions to transport. The effective mass tensors were calculated assuming a temperature of 300 K.^38^ We report an average of the three principal directions of transport (trace of the tensor divided by three). We do not report the conductivity effective mass for materials with a band gap less than 0.1 eV.^39^

### Bulk Sample Synthesis and XRD Characterization

Powder syntheses of BaCd_2_P_2_, CaCd_2_P_2_, SrCd_2_P_2_, BaCd_2_As_2_, and SrCd_2_Bi_2_ were attempted using elements in the stoichiometric 1:2:2 ratio (A:M:Pn). See SI Section 1 for safety information for performing these syntheses. Elemental Ba (99.9%, Alfa Aesar), Ca (99.98%, Alfa Aesar), Sr (99.90%, Sigma-Aldrich), Cd (99.95%, Alfa Aesar), red phosphorus (98.90%, Alfa Aesar), As (99.999995%, Furukawa Denshi Co.), and Bi (99.998%, Alfa Aesar) were used for their respective reactions. The reaction mixtures were placed into carbonized 9/11 mm inner/outer diameter silica ampules under an Ar atmosphere in a glovebox. The ampules were evacuated and then sealed using a hydrogen–oxygen torch. The sealed ampules were heated in a muffle furnace to a temperature ramp up to 800–1000 °C over 8–10 h and annealed at that temperature for 48 h. After cooling in the turned-off furnace, the ampules were then opened in ambient conditions. The resulting samples were ground in agate mortars and reannealed at 800–1000 °C for 48 h in an evacuated carbonized silica ampule. The synthesized powder samples were characterized by powder X-ray diffraction (PXRD). PXRD patterns were collected using a Rigaku MiniFlex 600 benchtop diffractometer with Cu Kα radiation (λ = 1.5406 Å) and Ni Kβ filter in the 3° ≤ 2θ ≤ 90° range with 0.02° steps at 10°/min. Four samples, BaCd_2_P_2_, CaCd_2_P_2_, SrCd_2_P_2_, and BaCd_2_As_2_, which were single-phase according to powder X-ray diffraction, were used for subsequent analyses. The SrCd_2_Bi_2_ sample has no target phase but rather a mixture of SrCdBi_2_ + Cd. See Figure S5 for PXRD patterns of the compounds investigated here and Table S4 for their lattice parameters.

### Thin Film Sample Synthesis and XRD Characterization

Thin films of CaZn_2_P_2_ and SrZn_2_P_2_ were synthesized by radio frequency (RF) cosputtering with 50.8 mm diameter metallic Ca, Sr, and Zn targets. The RF power densities applied to the targets were maintained in the range 1.48–1.97 W cm^–2^ for Ca, 1.13–1.25 W cm^–2^ for Sr, and 2.96 W cm^–2^ for Zn. The base pressure of the growth chamber was maintained below 10^–7^ Torr prior to deposition. The deposition was conducted in a 2% PH_3_/98% Ar gas mixture at a flow rate of 19.5 sccm, with the process pressure maintained at 5 mTorr through a throttled gate valve. PH_3_ is highly toxic and pyrophoric, and residual phosphorus deposits in the growth chamber can spontaneously ignite. Therefore, an O_2_ purging step was implemented after each growth to remove any unreacted phosphorus from the growth plate before transferring the sample to the loadlock. As a result, traces of O incorporation in the films may be anticipated. The safety requirements and precautionary procedures for working with a PH_3_ chamber are explained in a previous article^22^ and in SI Section 1. Compositionally uniform films were achieved by rotating the plate during growth. Crystalline CaZn_2_P_2_ and SrZn_2_P_2_ thin films were grown on a-SiO_2_ (fused silica) at 200 °C with a deposition time of 2 h, yielding a thickness of ∼500 nm. The crystalline nature of the films was confirmed via high-resolution synchrotron grazing incidence with wide-angle X-ray scattering (GIWAXS). Measurements were performed at beamline 11–3 at Stanford Synchrotron Radiation Lightsource. A Rayonix 225 area detector was used to collect data at room temperature using an X-ray wavelength of λ = 0.97625Å and an incident angle of 3°. See Figure S5 for PXRD patterns of the compounds investigated here.

### Photoluminescence (PL) Analysis

Synthesized powdered samples of BaCd_2_P_2_, CaCd_2_P_2_, SrCd_2_P_2_, and BaCd_2_As_2_ were compressed into pellets for PL analysis. Additionally, PL data were collected from SrZn_2_P_2_ and CaZn_2_P_2_ thin films, ∼500 nm thick, grown on a-SiO_2_ substrates. PL spectra were recorded using a Horiba Labram HR Evolution Raman spectrometer equipped with a micro-PL setup. The measurements employed a 50× objective lens and a 300 gr/mm grating, conducted at a temperature of 298 K to probe the band-to-band transitions for these materials. A 532 nm (∼2.33 eV) laser source with a power output of 100 mW, attenuated by a 1% neutral-density (ND) filter, was used. Considering that the optical efficiency of the equipment was determined to be ∼30%, the effective laser power at the sample was approximately 0.3 mW. PL spectra were collected within the spectral range of 650–1050 nm (1.91–1.18 eV) for the BaCd_2_P_2_, CaCd_2_P_2_, and SrCd_2_P_2_ samples. For the BaCd_2_As_2_ sample, the spectral range was extended to 1100–1800 nm (1.12–0.69 eV) to accommodate its lower energy band transitions by using InGaAs charge-couple device (CCD) detectors. For the SrZn_2_P_2_ and CaZn_2_P_2_ thin films, measurements were performed in the same spectral range as that of the powdered samples to facilitate comparison. To ensure the reliability of the data and mitigate site-specific variations, multiple regions on each sample were measured.

## Results and Discussion

Despite the growing interest in the AM_2_Pn_2_ Zintl compounds, not all elemental combinations have been systematically explored. Isoelectronic mutation with A = Ca, Sr, Ba, Yb, Mg; M = Zn, Cd, Mg; and Pn = N, P, As, Sb, Bi amounts to 75 total possible compositions. We note that some Eu-containing AM_2_Pn_2_ compounds have been experimentally reported, but we have chosen to exclude these from our investigation due to convergence issues with Eu.^40–44^ Querying the Materials Project for AM_2_Pn_2_ returns 56 AM_2_Pn_2_ entries in five unique crystal structure prototypes, with space groups *P*3̅*m*1, *I*4/*mmm, Pnma, I*4/*mcm,* and *C*2/*m.* Upon further inspection, the *I*4/*mcm* and *C*2/*m* are entirely hypothetical for the AM_2_Pn_2_ with no experiments confirming their existence, so they will be excluded from further consideration. Compounds with the same stoichiometry as AM_2_Pn_2_ have been confirmed in the *R*3̅*m* and P4/*mmm* structure type (such as NaSn_2_As_2_ ^45^ and BaPt_2_P_2_,^46^ respectively); however, they all contain elements beyond those considered here and do not appear within the AM_2_Pn_2_, so we exclude them as well. The *P*3̅*m*1, *I*4/*mmm*, and *Pnma* structure prototypes are shown in Figure 1. Of those 56, 39 of the AM_2_Pn_2_ reported on the Materials Project are in the *P*3̅*m*1 space group. Here, we report on our stability analysis and band structure for all of these potential compositions.

Figure 2 shows the thermodynamic stability, described by the hull energy, of AM_2_Pn_2_ compounds. The color scale is *E*_hull_ as shown in Equation 2; negative values represent that the AM_2_Pn_2_ is stable, whereas positive values represent thermodynamically favorable decomposition into competing phases. The data points shaped as circles indicate that the ground-state structure for AM_2_Pn_2_ is found to be the *P*3̅*m*1 structure, while the square data points correspond to a ground state in one of the other structure types found from the Materials Project shown in Figure 1. See Figure S3 for full details of the relative stability of other types. From Figure 1, it is visible that a large portion of possible compositions are predicted to be stable for isoelectronic substitution across the A and M sites. In addition, most of the AM_2_Pn_2_ do favor the *P*3̅*m*1 space group. The exceptions to these are AM_2_N_2_, where only a few AMg_2_N_2_ and AZn_2_N_2_ are stable. The compounds with the cation A site occupied by Mg are mostly unstable, despite being stable in well-characterized thermoelectric materials such as Mg_3_Sb_2_ (or MgMg_2_Sb_2_) and Mg_3_Bi_2_ (MgMg_2_Bi_2_). In our screening, we additionally evaluate the binary Mg_3_Pn_2_ in the *Ia*3̅ space group structure since this was also reported on the Materials Project, but does not have a ternary analog. We note that Mg_3_Sb_2_ has been identified to be close to a dynamical instability due to the size mismatch between Mg and Sb in the *P*3̅*m*1 space group.^48^ The ground-state structure of BaZn_2_Pn_2_ is not *P*3̅*m*1, which is understood from the large size difference between the A and M sites in these two compounds.^49^

**Figure 2:**
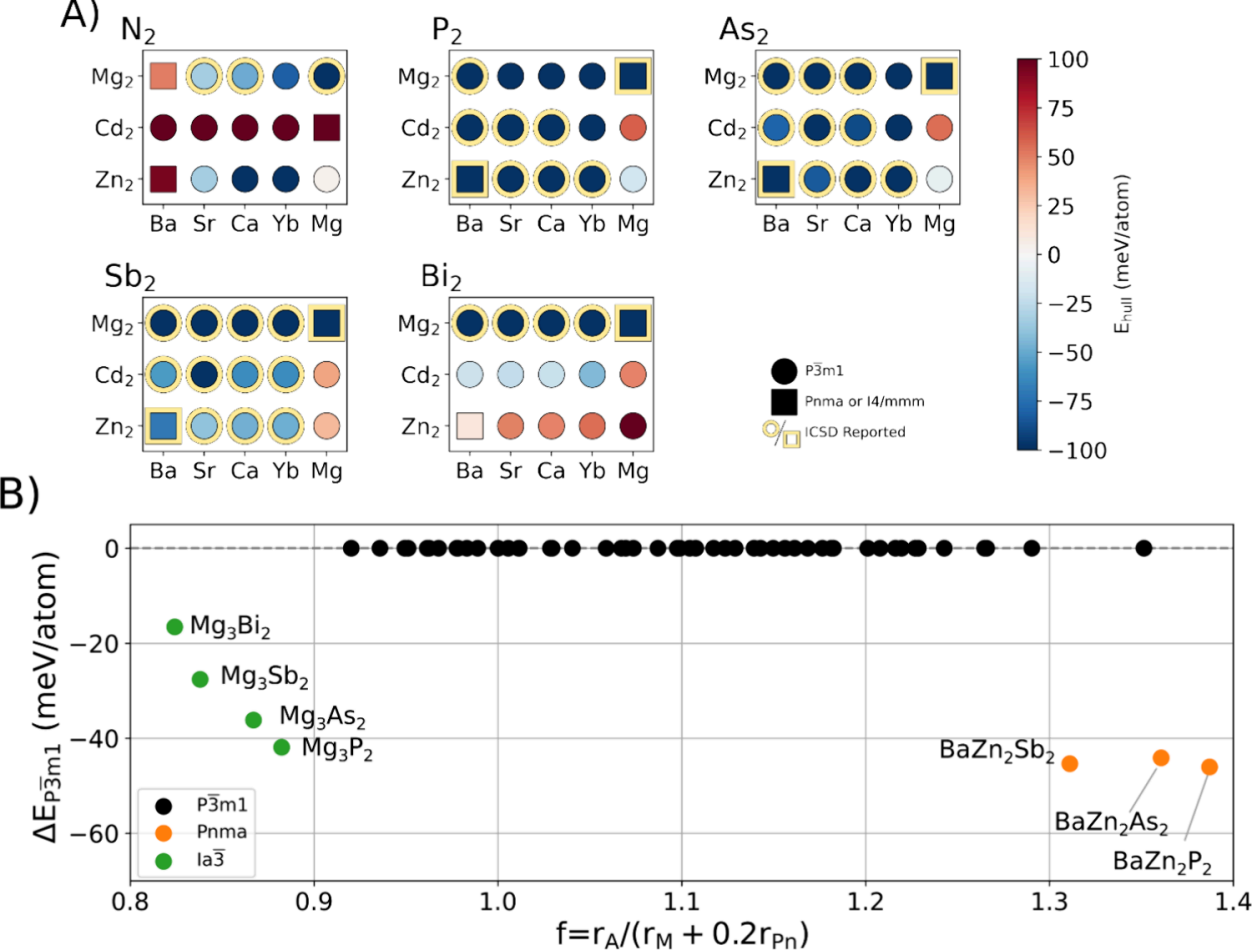
(A) Stability of the AM_2_Pn_2_. Matrix showing the r^2^SCAN predicted energies for the formation of each AM_2_Pn_2_ from its decomposition products. Here, a negative *E*_hull_ (blue) indicates stability and a positive (red) indicates instability. Circular data points represent compositions where *P*3̅*m*1 is the ground-state structure, and squares represent cases where the ground state is one of the other two structures presented in Figure 1. Compositions reported in ICSD 5.3.0 (ICSD release 20241001–1145) are bordered in yellow. See Table S1 for tabulated data from this plot. (B) Empirical size correlation of Klüfers and Mewis^50^ versus calculated energy difference between the most stable polymorph and the *P*3̅*m*1 polymorph, Δ*E*_*P*3̅*m*1_, for stable compounds only (*E*_hull_ < 0). A plot of all compounds in this investigation is in Figure S2. Color of the point represents the ground-state space group of the composition. For clarity, text labels of the *P*3̅*m*1 phases have been removed.

In Figure 2b, we compare our calculated data to the empirical stability rule based on atomic radii proposed by Klüfers and Mewis:^50^

$$f=\frac{r_{A}}{r_{M}+0.2r_{Pn}}$$ 5

We find that our data match the trend of Peng et al.^49^ in that there is an approximate threshold of *f* ≳ 1.25 above which the *P*3̅*m*1 structure type is less likely to be stable compared to *I*4/*mmm* or *Pnma* structure types. We additionally included the Mg_3_Pn_2_ compositions, which allows access to lower *f* values and shows that there is a lower threshold for *f* ≲ 0.9 where the *Ia*3̅ structure type is stabilized over *P*3̅*m*1 at 0 K. In Peng et al.,^48^ it is noted that *P*3̅*m*1 Mg_3_Sb_2_ is close to a dynamical instability, which gives this phase a high vibrational entropy. The proximity of Mg_3_Sb_2_ to the threshold further rationalizes these results. This is a relatively wide window of *f* for which *P*3̅*m*1 is favored, explaining why the majority of phases favor formation in this structure. The implications of Equation 5 are that the pnictogen ion has a relatively small impact on the structural stability of the AM_2_Pn_2_. For a combination of the largest A ion, Ba, and relatively small Zn, *f* increases above the threshold, leading to the stabilization of other structure types.

Among the 75 compositions investigated, 40 are recorded in the ICSD.^51^ Thirty-seven out of these 40 experimentally observed compounds match the ICSD records in terms of the existence of a compound of that composition (showing its stability) and the compound’s reported space group, demonstrating the predictive power of our approach. Mg_3_Sb_2_, Mg_3_Bi_2_, and Mg_3_N_2_ are all correctly predicted to be stable, but not in the correct space groups. Mg_3_Sb_2_ and Mg_3_Bi_2_ are predicted to crystallize in the *Ia*3̅ space group structure, whereas they are reported in the *P*3̅*m*1 space group structure. The *Ia*3̅ space group structure is 28 and 17 meV/atom lower in energy than the *P*3̅*m*1 space group structure for Mg_3_Sb_2_ and Mg_3_Bi_2_, respectively. Testing Mg_3_Sb_2_ with PBEsol correctly predicts the *P*3̅*m*1 space group structure as the ground-state structure. See Tables S6 and S7 for a comparison of the lattice parameters and energy differences. The calculation for Mg_3_N_2_ in the *Ia*3̅ space group structure did not converge, so its stability could not be predicted.

There are a number of compounds we predict to be stable that are not found in the ICSD. In the literature, some of these have actually already been synthesized experimentally with reported PXRD patterns, including SrZn_2_N_2_,^52^ CaZn_2_N_2_,^53,54^ MgZn_2_P_2_,^55^ and YbCd_2_As_2_.^56^ The remaining compounds are new compounds not synthesized yet but should be synthesizable (*E*_hull_ < 0) according to our calculations: YbMg_2_N_2_, YbZn_2_N_2_, CaMg_2_P_2_, SrMg_2_P_2_, YbMg_2_P_2_, YbCd_2_P_2_, YbMg_2_As_2_, MgZn_2_As_2_, BaCd_2_Bi_2_, CaCd_2_Bi_2_, SrCd_2_Bi_2_, and YbCd_2_Bi_2_. To our knowledge, SrMg_2_P_2_,^57^ CaMg_2_P_2_,^58^ BaCd_2_Bi_2_,^59^ CaCd_2_Bi_2_,^60^ and SrCd_2_Bi_2_ ^61^ have previously been studied computationally.

For the rest of our discussion focusing on the electronic structure, we will only consider the AM_2_Pn_2_ composition in the *P*3̅*m*1 structure type because the majority of compounds are stable in this structure type.^58,65–70^ Figure 3 shows the band gaps for the *P*3̅*m*1 AM_2_Pn_2_ compounds. Relatively few of them have experimentally reported band gaps. We note that we computed the band gaps using HSE + SOC based on the PBE-relaxed structures. In order to show the trend of the band gap with composition more clearly, we have chosen to include band gaps of all compositions in the same *P*3̅*m*1 structure type. We have noted which compounds are stable in the *P*3̅*m*1 structure type with a gray ring around the data point. Figure 3 shows that the *P*3̅*m*1 AM_2_Pn_2_ materials have a wide range of band gaps, spanning from 0 to over 3 eV. A table of the band gaps for AM_2_Pn_2_ compounds is available in Supplemental Table S2, and full band structures can be found in Figure S4. As commonly observed, the band gaps decrease with moving down the pnictogen group from nitrides to bismuthides. For a given anionic metal, Mg on the M site has the largest band gap. Zn- and Cd-containing analogs often have similar band gaps, but Cd more often has a direct band gap and Zn is usually indirect. The band gap is not strongly affected by the element on the A site. The wide range of band gaps means a wide range of potential applications. The AM_2_Pn_2_ materials are already recognized as promising thermoelectric materials, but this range of band gaps and the recent results on the defect tolerance of BaCd_2_P_2_ and CaZn_2_P_2_ suggest they can also make promising infrared detectors, single-junction solar cells, tandem solar top cells, light-emitting diodes (LEDs), or photoelectrocatalysts.^21,22^ In a later section, we will discuss how candidate compounds can be used in specific applications.

**Figure 3:**
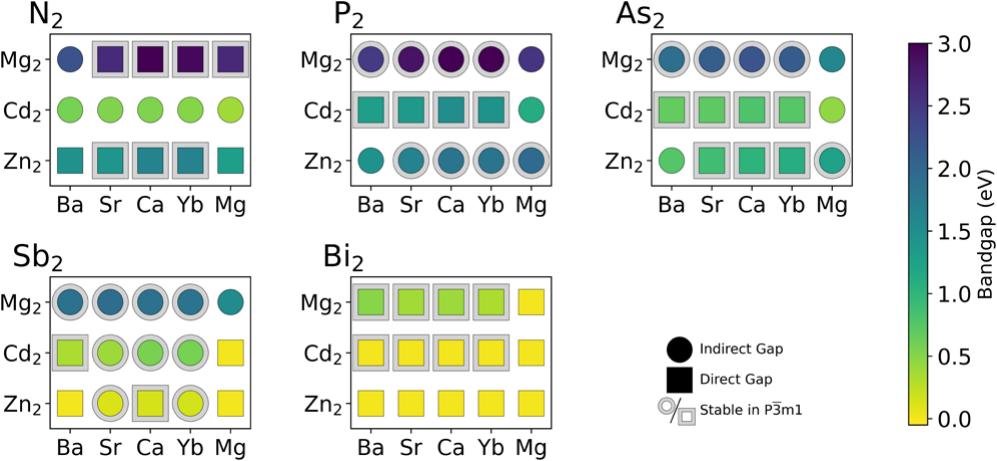
HSE +SOC band gaps of AM_2_Pn_2_ in the *P*3̅*m*1 structure. The data point shape denotes the nature of the band gap, squares are direct band gap materials, and circles are indirect. See Figure S4 for full band structures. Compositions stable (*E*_hull_ < 0) in the *P*3̅*m*1 space group structure are bordered in gray.

Rather than simply categorizing each material as direct or indirect, we have quantified the difference between these band gaps, Δ*E*_*g*_, shown in Figure 4. When considering potential applications for materials, especially in optoelectronics, those with an indirect band gap are not optimal, but a small Δ*E*_*g*_ can be tolerable.^62^ For example, in a thin film solar absorber, the optical absorption is primarily due to direct band-to-band transitions, whereas the open circuit voltage, *V*_oc_ is always less than the minimum band gap, so a Δ*E*_*g*_ > 0 represents a decrease in optical absorption for a given *V*_oc_. Twelve materials in this study have 0 < Δ*E*_*g*_ < 0.1 eV, meaning they are somewhat close to a direct material. Of these nearly direct materials, Mg_3_As_2_ has the largest indirect band gap (1.49 eV) while all other nearly direct materials have band gaps less than 0.7 eV. Mg_3_Sb_2_ is the most indirect material, with a 0.55 eV indirect band gap and a 1.54 eV direct band gap. The subfamily SrMg_2_P_2_, YbMg_2_P_2_, and CaMg_2_P_2_ are also highly indirect with Δ*E*_*g*_ = 0.72, 0.93, and 0.89 eV, respectively.

**Figure 4:**
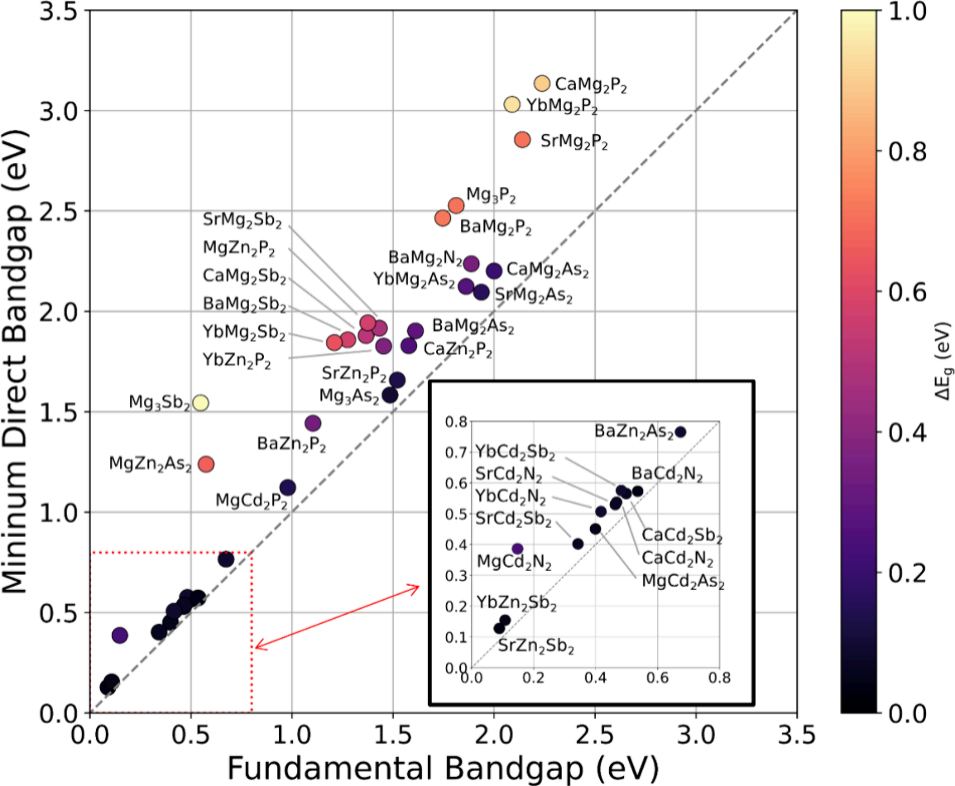
Fundamental vs minimum band gap of the *P*3̅*m*1 AM_2_Pn_2_ materials. The color scale is the difference between the direct and indirect band gap, Δ*E*_*g*_. Direct band gap materials are omitted because they have Δ*E*_*g*_ = 0 by definition. Small band gap materials are shown as inset for clarity. The *x* = *y* line (gray, dashed) is shown as a visual reference.

From our band structure calculations, we also extracted the estimated average carrier conductivity effective masses for the *P*3̅*m*1 AM_2_Pn_2_ materials. Effective masses set carrier mobility, which is important in PV or thermoelectric applications, where low effective masses are desirable for both applications. From Figure 5a, it is seen that the class of materials studied here has relatively low electron masses, typically below about 1.0 m*_e_, and about 0.5 m*_e_ on average. Some exceptions to this are BaZn_2_As_2_, SrZn_2_As_2_, BaCd_2_Sb_2_, and SrZn_2_Sb_2_, which have much higher electron effective masses. On the other end of the spectrum, the nitrides have quite low effective masses, less than 0.25 m*_e_ on average. For the cation M site, Cd generally leads to lower electron effective masses than Mg or Zn counterparts. For holes, there is more variation in the effective mass throughout the family. Figure 5b shows that nitrides have the heaviest holes. Other pnictides have a fairly consistent hole effective mass of ∼0.75 m*_e_. We note that for thermoelectric applications, it is the majority carrier transport that is most important, while photovoltaics, which involve optical excitations, tend to depend on the minority carrier transport.

**Figure 5:**
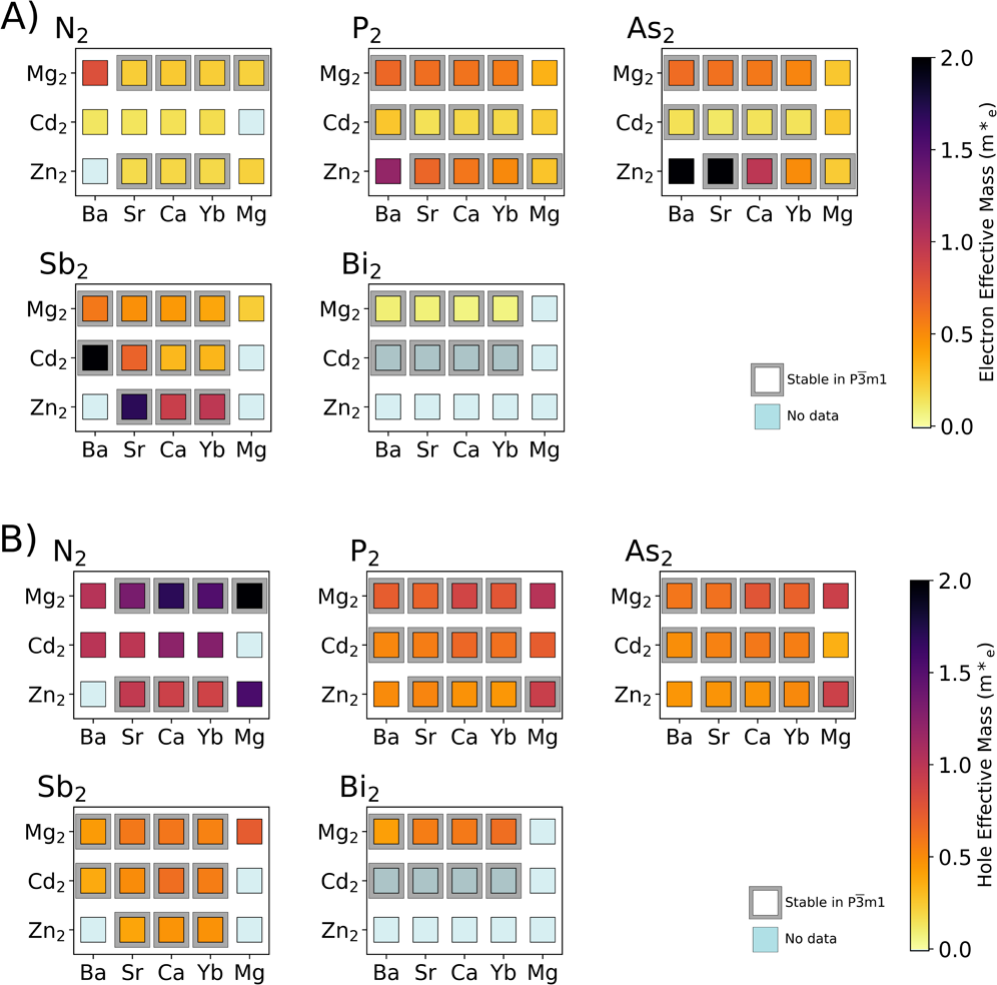
Average conductivity effective masses of (A) electrons and (B) holes for the *P*3̅*m*1 AM_2_Pn_2_. Materials with computed band gaps less than 0.1 eV have been excluded. Light blue represents no data, either because of exclusion or because of no completed calculations. Compositions stable (*E*_hull_ < 0) in the *P*3̅*m*1 space group are bordered in gray.

Here, we compare our calculated band gaps to experimental data available. We note that the HSE + SOC band gaps are based on PBE-relaxed structures. Overall, we find excellent agreement between the calculated band gaps and the experimental reports, as shown in Figure 6. Excluding specific measurements for CaZn_2_P_2_,^63^ SrCd_2_As_2_,^64^ and BaCd_2_Sb_2_ ^65^ (discussed below), the mean average error (MAE) is 0.16 eV. This is consistent with other reports on the agreement between full HSE calculations and experimental band gaps.^66,67^ This gives us confidence in our results and that they can be used predictively to apply to the materials not yet well characterized. The most overestimated band gap is CaMg_2_Bi_2_, which we calculate is 0.40 eV direct band gap but is reported to be 0.2 eV by electrical resistivity measurements by May et al.,^68^ although this measurement is likely a lower limit for the band gap since the technique used may be measuring the transition between the valence band and a midgap defect state if present. Conversely, the most underestimated band gap is SrCd_2_Sb_2_, which we predict should have an indirect band gap of 0.34 eV and a minimum direct band gap of 0.40 eV, but it is measured as 0.63 eV by Jin et al.^69^ with optical measurements on a Tauc plot. So, in general, our results match experiments well and should be applicable to predictions for those systems that have not yet been investigated and reported experimentally.

**Figure 6:**
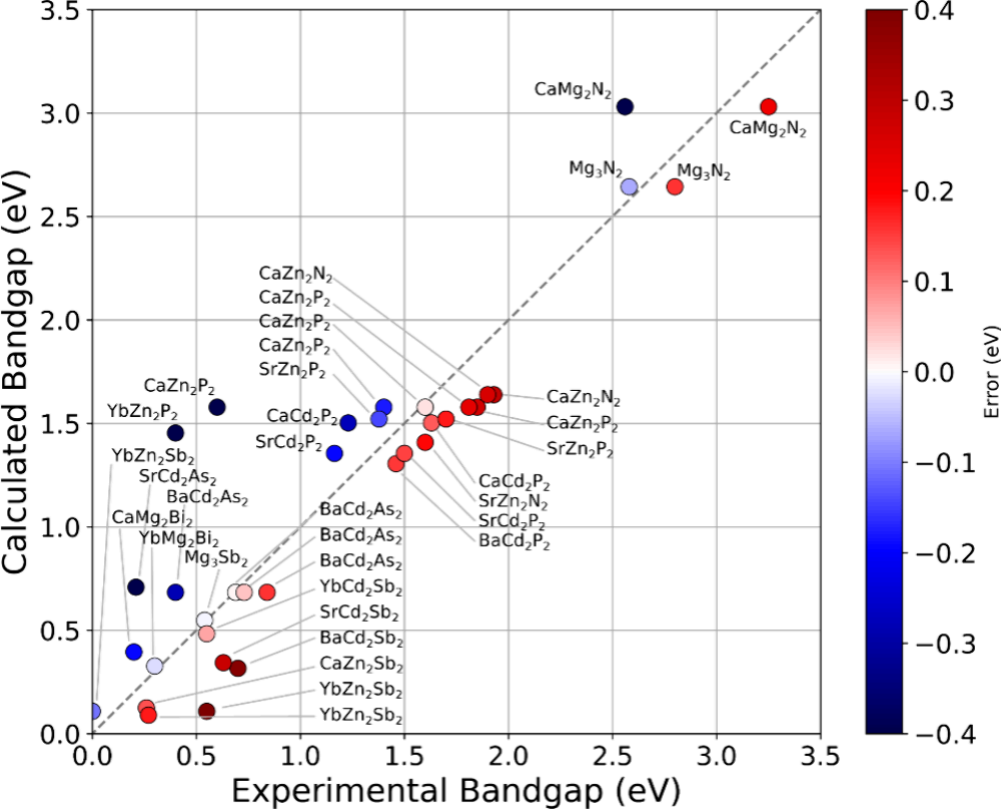
Comparison of experimentally reported band gaps and our calculations. The color of the point represents the difference between the two values. The *x* = *y* line (gray, dashed) is shown as a visual reference.

As mentioned previously, we find several reports that neither match our calculations nor other published band gap values. We summarize those findings here and present a full discussion in SI Section 3. In the case of CaZn_2_P_2_, there have already been several independent measurements of its band gap, so by direct comparison, we can eliminate reports by Ponnambalam et al. of a 0.6 eV band gap.^63^ It is still uncommon to have several band gap measurements of the same material in this class of compounds, but by looking at the trends in band gap in groups of similar compounds, we notice several inconsistencies. For example, Chen et al.^64^ measured a 0.21 eV band gap for SrCd_2_As_2_, which is less than reports of the band gaps for BaCd_2_As_2_ and SrCd_2_Sb_2_, though it is expected to be higher than either of these from our calculations, as well as their trend in atomic mass. It appears our calculations underestimate the band gap of CaZn_2_N_2_ by 0.27 eV, likely due to overestimation of the lattice parameter from the initial PBE relaxation. Fully HSE calculations can correct this error but at an increased computational cost.

### Experimental Verification

We have synthesized BaCd_2_P_2_, CaCd_2_P_2_, SrCd_2_P_2_, and BaCd_2_As_2_ powders and CaZn_2_P_2_ and SrZn_2_P_2_ thin films and performed photoluminescence spectroscopy studies, shown in Figure 7, to obtain their band gaps. The measured (calculated) direct band gaps for BaCd_2_P_2_, CaCd_2_P_2_, SrCd_2_P_2_, BaCd_2_As_2_, CaZn_2_P_2_ and SrZn_2_P_2_ are 1.46 eV (1.31 eV), 1.58 eV (1.50 eV), 1.29 eV (1.35 eV), 0.73 eV (0.68 eV), 1.93 eV (1.83 eV), and 1.81 eV (1.66 eV), respectively. For BaCd_2_P_2_, CaCd_2_P_2_, SrCd_2_P_2_, and BaCd_2_As_2_, we have assigned the highest peak to the band-to-band transition, though BaCd_2_P_2_ does show a low-intensity sub-band gap defect peak at about 1.25 eV. It was also recently shown that the band gap of BaCd_2_P_2_ quantum dots blue shifts under size confinement, increasing up to 1.8 eV in ca. 2 nm nanocrystals based on the shifting of the photoluminescence peak.^70^ For CaZn_2_P_2_, we have assigned the highest peak to the band-to-band transition corresponding to the direct band gap. We attribute the second smaller peak centered at 1.65 eV to CaZn_2_P_2_’s indirect band gap, which we calculate to be 1.58 eV. These experimentally measured band gap values agree well with our computational results, despite the slight systematic underestimation due to the inaccuracy of the lattice parameters expected from the PBE relaxation. To the best of our knowledge, our results are the first report of the experimentally measured band gap for CaCd_2_P_2_ and SrCd_2_P_2_.

**Figure 7:**
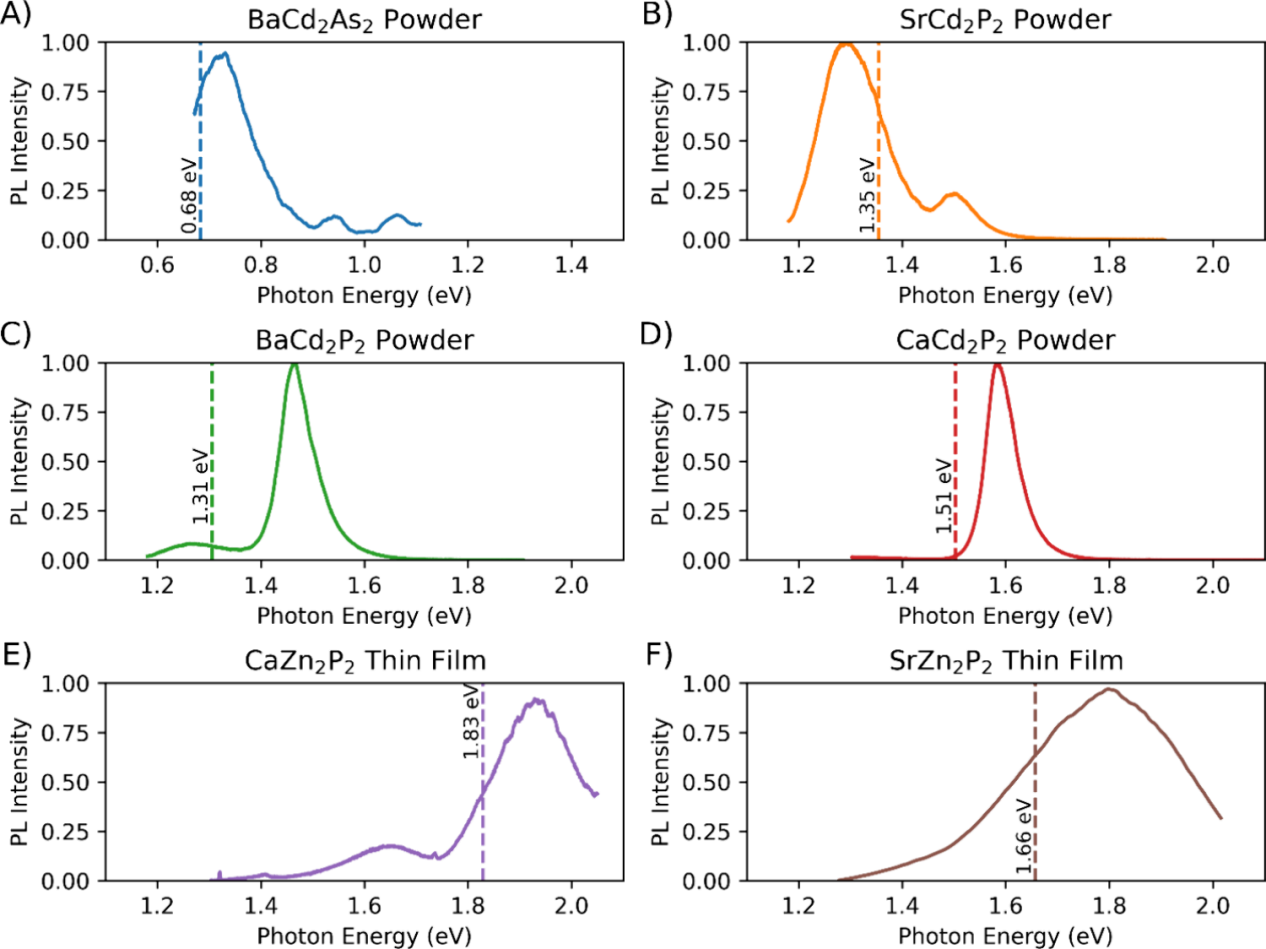
PL spectra of select AM_2_Pn_2_. (A–D) powder samples of BaCd_2_As_2_, SrCd_2_P_2_, BaCd_2_P_2_, and CaCd_2_P_2_, respectively. (E, F) Thin film samples of CaZn_2_P_2_ and SrZn_2_P_2_. Vertical dashed lines represent the HSE + SOC calculated direct band gaps for visual reference to compare between experiment and computations. PL spectrum of CaZn_2_P_2_ is taken from Quadir et al.^22^

There are conflicting experimental reports of the band gap of BaCd_2_As_2_. In Kunioka et al.,^71^ the band gap is reported to be 0.40 eV, while Yang et al.^72^ report that their measurement of the band gap is more than double that, at 0.84 eV. Both measurements similarly estimate the band gap from the change in resistivity with respect to temperature. Our calculation of 0.68 eV more closely matches Yang et al.,^72^ but is close to the middle of the two measurements, so it makes it difficult to discern which report is more accurate. Now, with our optical data, we report that our experimental measurement of the band gap is 0.73 eV, which matches very closely with our calculation and agrees better with the band gap reported in Yang et al.^72^ This clarifies the band gap of BaCd_2_As_2_ with greater accuracy.

To verify our prediction of new (i.e., previously unreported) compounds, we have tried to synthesize SrCd_2_Bi_2_. From Figure 2, the ACd_2_Bi_2_ compounds are predicted to be stable with an *E*_hull_ that is only slightly negative, yet they have not been reported. Unfortunately, our solid-state reactions do not result in the target compound but instead SrCdBi_2_ + Cd. This can be rationalized given the small *E*_hull_ (−24 meV/atom) for SrCd_2_Bi_2_, which has a predicted decomposition of SrCdBi_2_ + Cd. In chemical synthesis, the formation of target compounds with small decomposition energies can be kinetically controlled to form other metastable products.^73^ We have not tested alternate synthetic routes for SrCd_2_Bi_2_, such as starting from binary precursors rather than elemental precursors, which may still allow the formation of this new compound.

### Application-Specific Candidate Selection

The electronic structure of AM_2_Pn_2_ can be widely tuned by varying chemistry. We now discuss what chemistries could work best for certain applications, including thermoelectric devices, infrared detectors, and photovoltaics.

The most widespread application considered for AM_2_Pn_2_ compounds is thermoelectric materials. Thermoelectric applications favor small band gap semiconductors, as it correlates with the potential for high doping and electrical conductivity. It also requires low lattice thermal conductivity, which is easier to reach with heavier elements. In that respect, the antimonides present the most adequate chemistry and they have indeed been widely studied in the thermoelectric field with EuZn_2_Sb_2_,^74^ YbCd_2–x_Zn_*x*_Sb_2_,^75^ and most notably with Mg_3_Sb_2_.^17,19,20,76^ Another desirable property for thermoelectric materials is low effective masses combined with valley degeneracy. Valley degeneracy is enhanced by conduction or valence band pockets off the Γ point due to their higher multiplicity in the Brillouin zone.^77^ The valence bands are centered in Γ for all of the antimonides, indicating that the electronic structure of a p-type thermoelectric in AM_2_Pn_2_ will not favor a high zT even if doping is achievable. On the other hand, the conduction band can have off-Γ pockets. Mg_3_Sb_2_ clearly has the highest valley degeneracy of all AM_2_Pn_2_ compounds with conduction band pockets at the *M*, *K*, and *L* points (Figure S4). Other AMg_2_Sb_2_ also have conduction band valley degeneracy, though to a lesser extent than Mg_3_Sb_2_. The effect of Mg on the band structure is to raise the conduction band at the Γ point, while keeping other local minima of the conduction band the same, as can be seen, for example, in the band structures of CaCd_2_Sb_2_ versus CaMg_2_Sb_2_ (see Figure S4). This matches the findings of Zhang et al.^20^ that, among the AM_2_Pn_2_, a multivalley conduction band is unique to the binary Mg_3_Pn_2_ compounds. These considerations do not account for whether it is actually possible to properly dope the material but just show that *n*-type doping is more promising than *p*-type doping.

Another emerging application for the AM_2_Pn_2_ is in PV. Notably, BaCd_2_P_2_ has shown a lot of potential as a single-junction solar absorber material.^21^ Defects, especially deep traps, can limit the performance of the solar absorbers. Defect computations and carrier lifetime measurements have shown favorable defect properties in the AM_2_Pn_2_ family, at least for BaCd_2_P_2_ and CaZn_2_P_2._ ^21,22^ Beyond single-junction materials, solar absorbers for tandem cells are of great interest. If combined with a silicon bottom cell with a band gap of 1.1 eV, a top cell with a band gap of ∼1.5–1.8 eV^78^ is most efficient. Our analysis shows a large range of phosphides, nitrides, and arsenides with suitable band gaps for single-junction or tandem solar cells. Many of the AMg_2_P_2_ or AMg_2_As_2_ have large band gaps, so they may seem promising, but we recognize that these compounds may not be air- or moisture-stable due to the Mg content.^79^ Fewer nitrides form the AM_2_Pn_2_ composition than other pnictogens, but Zn nitrides could be an option because of their suitable band gaps and a low electron effective mass. CaZn_2_N_2_ is especially appealing, as highlighted by previous reports^53,54^ which suggest its use as a solar absorber in a single-junction photovoltaic cell, though we find its band gap is better suited for tandem top cells. Although most high-efficiency Si, III–V, and perovskite cells use undoped or only lowly doped absorber layers, we note that this should be considered when selecting materials for solar cells. Computed defects in phosphides indicate that it will be very difficult to produce *n*-type BaCd_2_P_2_ and CaZn_2_P_2_. In general, AM_2_P_2_ compounds are likely to be insulators or *p*-type semiconductors. SrZn_2_N_2_ and CaZn_2_N_2_ are both weakly *n*-type semiconductors.^52,54^

The figure of merit for infrared detectors relates quite well to PV materials.^80,81^ Long carrier lifetime and high optical absorption are similarly sought after but in lower band gap materials. The favorable defect properties and absence of low formation energy and deep traps in phosphides could extend to smaller band gap materials such as antimonides. Interesting candidates include SrCd_2_Sb_2_ and CaZn_2_Sb_2_, both of which are thermodynamically stable and show a small direct band gap of 0.42 and 0.14 eV, respectively. Since the bismuthides form metallic systems with similar *P*3̅*m*1 structures, it could be attractive to alloy some of the isostructural antimonides with bismuth to tune the band gap to smaller values and make absorbers for long wavelength IR detection as performed in the Hg_1–*x*_Cd_*x*_Te (MCT) system.^82,83^

Finally, we note that given the broad stability of this class of materials and that most form in the same *P*3̅*m*1 structure, alloys of these various ordered compounds may form quite readily. This could open the way to tune the band gap and electronic structure to achieve the highest performance materials in thermoelectrics, photovoltaics, infrared detectors, and other applications.^76^

## Conclusions

The AM_2_Pn_2_ class of materials has a remarkable ability to form stable compounds in a large number of compositions, for which they are only just starting to have their full utility realized. In this work, we have computationally explored the family of AM_2_Pn_2_ Zintl compounds, composed of 75 compositions. We clarified their thermodynamic stability and equilibrium structure, showing that most of them are stable in the *P*3̅*m*1 space group. Twelve new stable AM_2_Pn_2_ compounds are predicted. We have studied the electronic band structures of *P*3̅*m*1 AM_2_Pn_2_ compounds, revealing that the band gap of this class of materials spans a wide range from metals to semiconductors above 3 eV. Given the stability and diversity of this compositional family, there may be a variety of applications for which these materials can be used, beyond the thermoelectric materials for which they are more commonly recognized. Based on the stability and electronic structure, we have selected preliminary candidates for tandem top cell solar absorbers, infrared detectors, and thermoelectric applications. We hope that this work serves as a roadmap to inspire more in-depth exploration of the individual AM_2_Pn_2_ compounds.

## Supplementary Material



## Notes

Safety warning, energy above hull from PBE, ground-state crystal structure study, tabulated thermodynamic data, tabulated bandstructure data, bandstructure plots, band gap comparison data selection, XRD, lattice parameter comparison, VASP pseudopotential versions, and functional comparison for Mg_3_Sb_2_ for lattice parameter and polymorph stability (PDF)
