# Supplementary material for: Map of the Zintl AM2Pn2 Compounds: Influence of Chemistry on Stability and Electronic Structure
Source: Chem Mater. 2025 Jun 24;37(13):4684–94. doi: 10.1021/acs.chemmater.5c00353 (PMC12243087; doi:10.1021/acs.chemmater.5c00353)
Supplement: Supplementary file 1 [file cm5c00353_si_001.pdf]

A Map of the Zintl AM_2Pn_2 Compounds: Influence of Chemistry on Stability and Electronic Structure

Andrew Pike[1], Zhenkun Yuan[1], Gideon Kassa[1], Muhammad Rubaiat Hasan[2], Smitakshi Goswami[1,3], Sita Dugu[4], Shaham Quadir[4], Andriy Zakutayev[4], Sage R. Bauers[4], Kirill Kovnir[2,5], Jifeng Liu[1], Geoffroy Hautier[1*]

1 Thayer School of Engineering, Dartmouth College, Hanover, NH 03755, USA

2 Department of Chemistry, Iowa State University, Ames, IA 50011, USA

3 Department of Physics and Astronomy, Dartmouth College, Hanover, NH 03755, USA

4 National Renewable Energy Laboratory, Golden, CO 80401, USA

5 Ames National Laboratory, U.S. Department of Energy, Ames, IA 50011, USA

*Corresponding Author: Geoffroy Hautier, geoffroy.hautier@dartmouth.edu

SI Section 1: Safety Warning

Warning: The starting reagent, alkaline-earth metals are air- and water-reactive and should be handled carefully in an inert atmosphere. At >400 °C inside the reaction ampoule, excessive vapor pressure of As or P as well as reaction of alkaline-earth metal with silica may compromise the silica ampoule resulting in shattering or explosion. The annealing steps must be conducted in a well-ventilated environment, such as in a fume hood.

Warning: We strongly emphasize that PH_3 is toxic and pyrophoric and P deposits leftover in a growth chamber can spontaneously combust during venting and routine chamber service (part changes, cleaning, etc.). Thus, additional safety controls, including robust interlocking, hydride gas monitoring, pump/purge cycling, self-contained breathing apparatus, flame-retardant personal-protective equipment, exhaust abatement, and others, must be rigorously implemented from the onset for any growth chamber intended to utilize PH_3 or prepare phosphide samples.

SI Section 2: Comparison of Stability calculated with PBE and r^2SCAN

With r^2SCAN, ACd_2Bi_2 are stabilized, moving them further below the hull whereas they were previously very nearly on the hull.

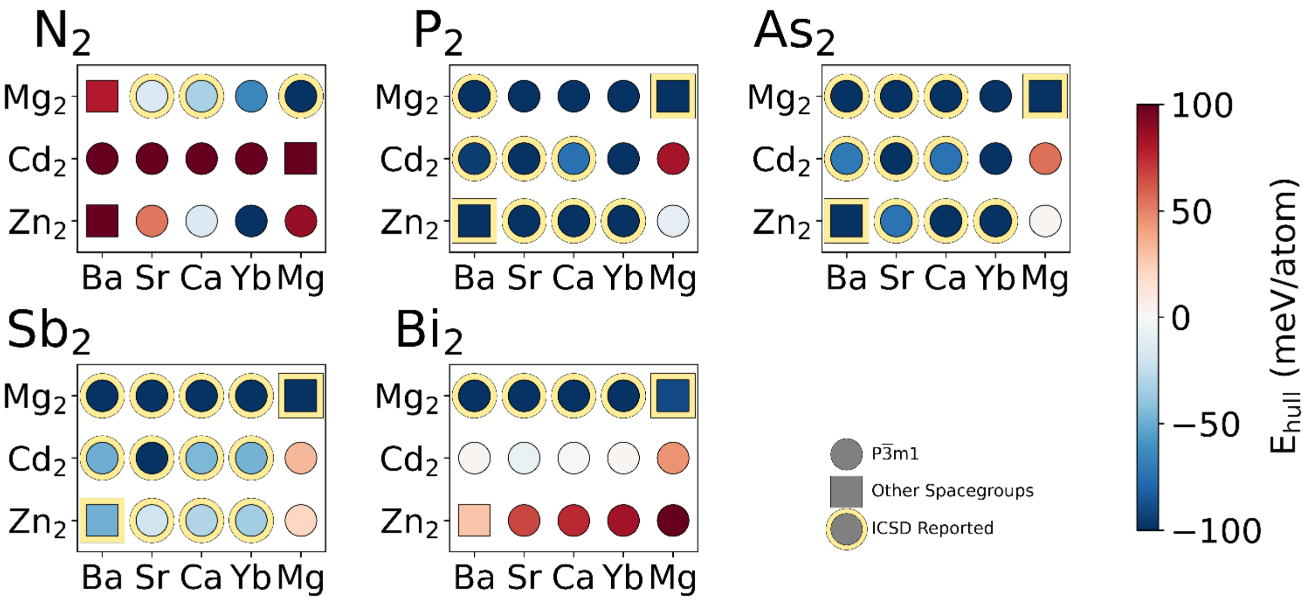

Fig. S1 - Stability of the AM_2Pn_2. Matrix showing the PBE predicted energies for each AM_2Pn_2 to decompose into its products. Here, a positive energy of decomposition (blue) indicates stability, and negative (red) indicates instability. Circular data points represent compositions where $P\bar{3}m1$ is the ground state structure, and squares represent where it is one other structure of the other four structures explored. Compositions reported on the ICSD are bordered in green.

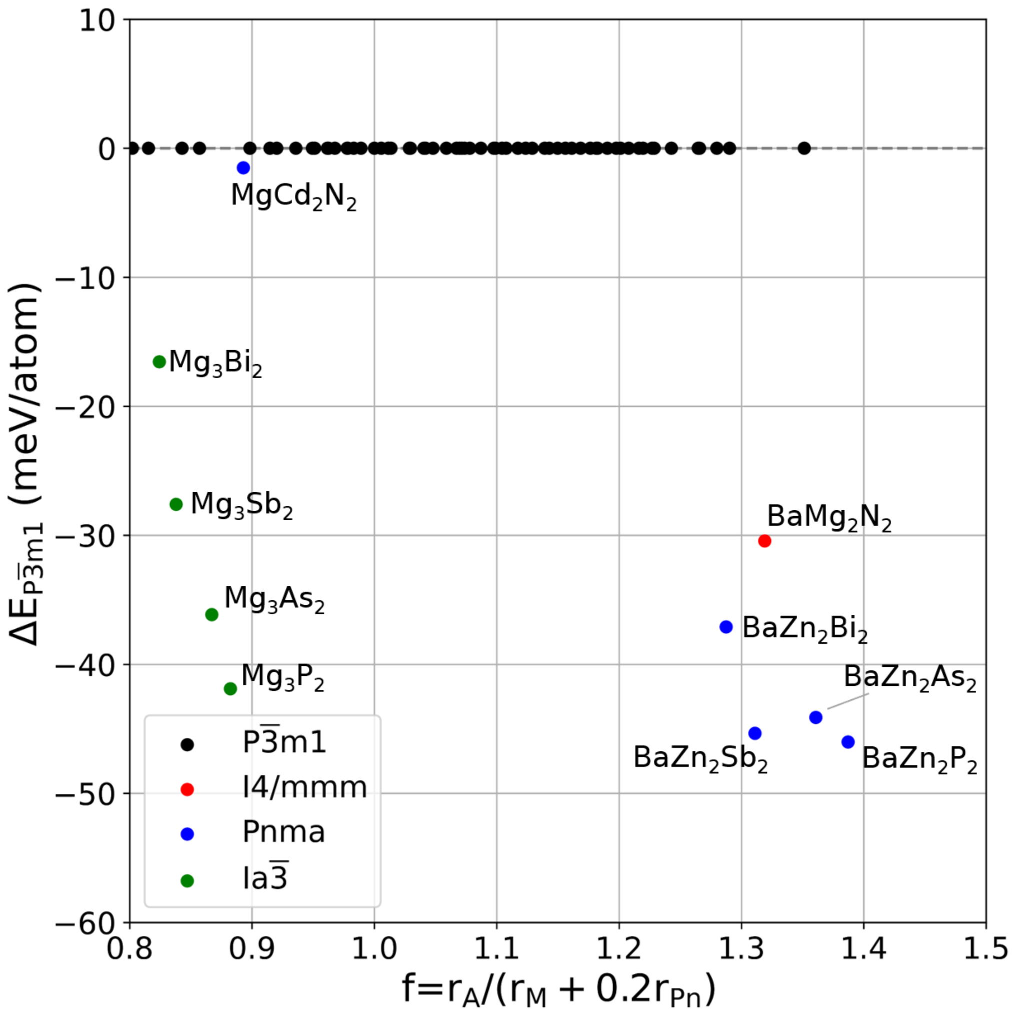

Fig. S2 - The empirical size correlation of Klufers et Al. [1] versus calculated energy difference between the most stable polymorph and the P-3m1 polymorph, $\Delta E_{P\bar{3}m1}$, for all compositions studied in this investigation. Color of the point represents the ground state space group of the composition. For clarity, text labels of the $P\bar{3}m1$ phases have been removed

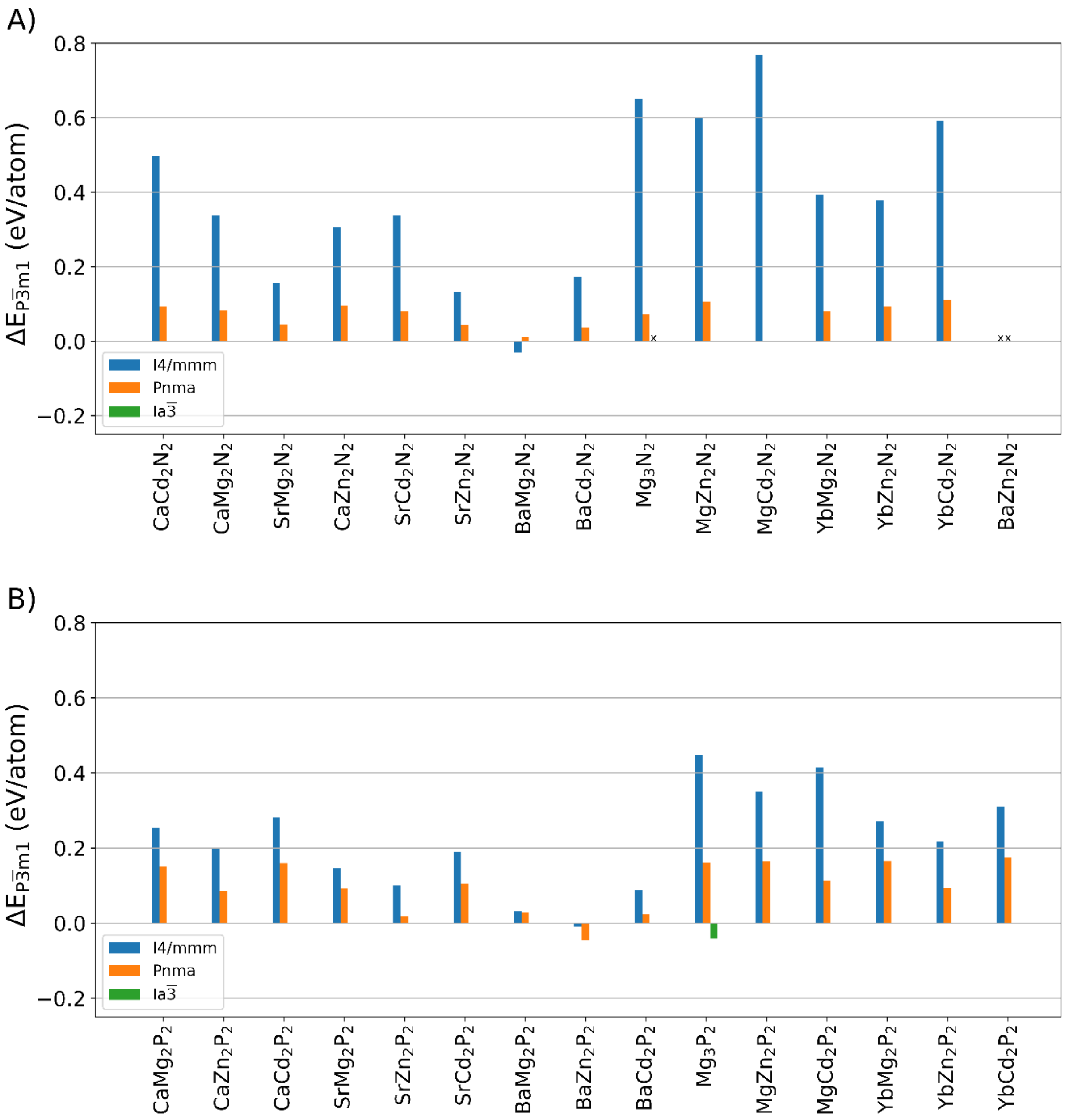
A)
B)
ΔE P3̄m1 (eV/atom)
I4/mmm
Pnma
Ia3̄
CaCd2N2
CaMg2N2
SrMg2N2
CaZn2N2
SrCd2N2
SrZn2N2
BaMg2N2
BaCd2N2
Mg3N2
MgZn2N2
MgCd2N2
YbMg2N2
YbZn2N2
YbCd2N2
BaZn2N2
CaMg2P2
CaZn2P2
CaCd2P2
SrMg2P2
SrZn2P2
SrCd2P2
BaMg2P2
BaZn2P2
BaCd2P2
Mg3P2
MgZn2P2
MgCd2P2
YbMg2P2
YbZn2P2
YbCd2P2

C)

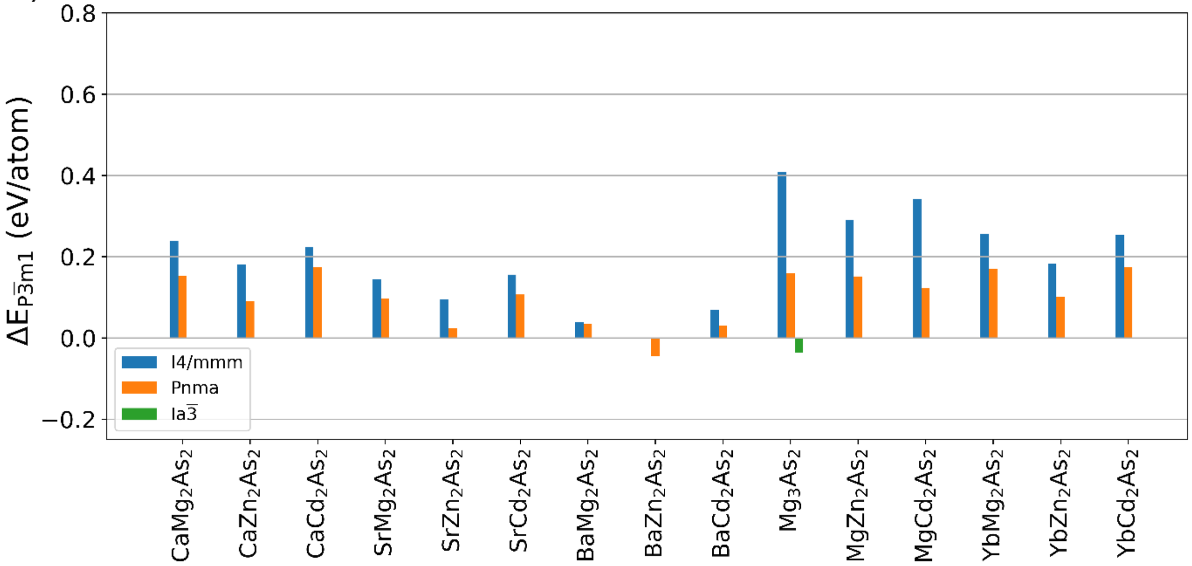

0.8
0.6
0.4
0.2
0.0
−0.2
ΔE_P3̄m1 (eV/atom)
I4/mmm
Pnma
Ia3̄
CaMg2As2
CaZn2As2
CaCd2As2
SrMg2As2
SrZn2As2
SrCd2As2
BaMg2As2
BaZn2As2
BaCd2As2
Mg3As2
MgZn2As2
MgCd2As2
YbMg2As2
YbZn2As2
YbCd2As2

D)

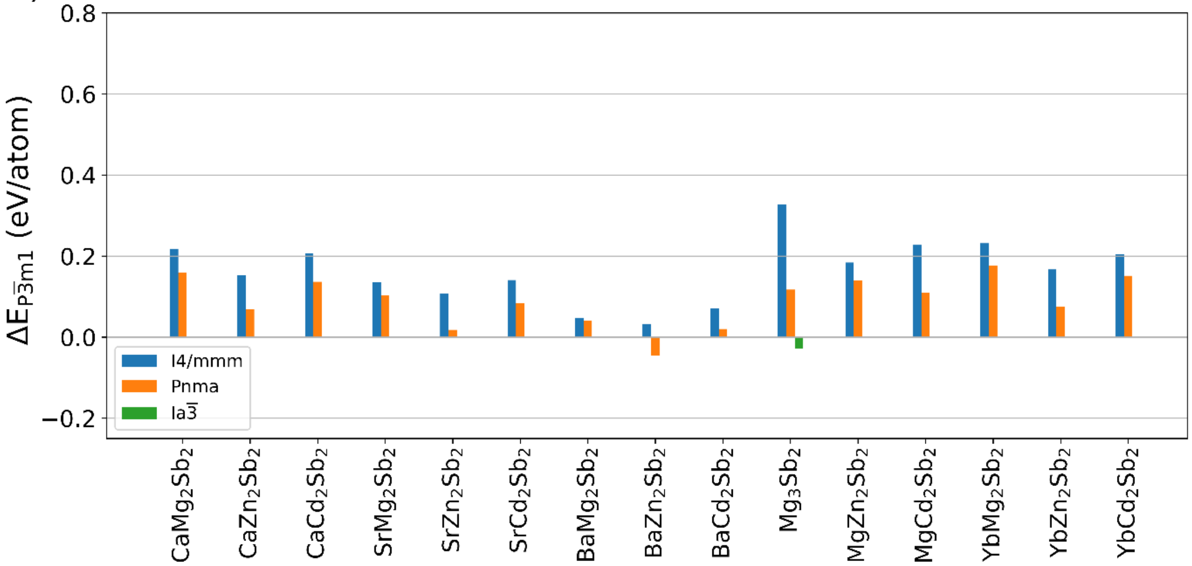

0.8
0.6
0.4
0.2
0.0
−0.2
ΔE_P3̄m1 (eV/atom)
I4/mmm
Pnma
Ia3̄
CaMg2Sb2
CaZn2Sb2
CaCd2Sb2
SrMg2Sb2
SrZn2Sb2
SrCd2Sb2
BaMg2Sb2
BaZn2Sb2
BaCd2Sb2
Mg3Sb2
MgZn2Sb2
MgCd2Sb2
YbMg2Sb2
YbZn2Sb2
YbCd2Sb2

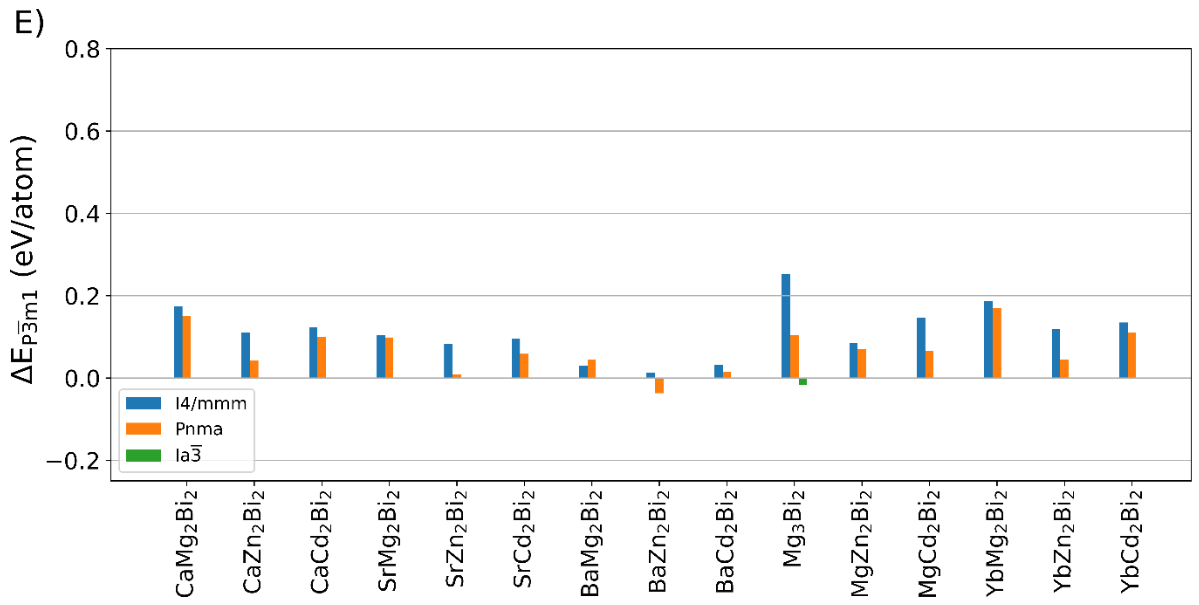

Fig. S3 – The relative stability of each AM_2Pn_2 compound. Here we use the final energy (eV/atom) of the $P\bar{3}m1$ polymorph as a reference to show to difference in energy of the other polymorphs. The plots are divided in A) nitrides B) phosphides C) arsenides D) antimonides and E) bismuthides

Table S1 - Stability information for AM_2Pn_2 compounds

Formula	Ground State Structure	Ehull (meV/atom)	Decomposition Products
**$BaCd_2As_2$**	$P\bar{3}m1$	-80	0.5 $Ba_2Cd_2As_3$ + 0.25 Cd_3As_2 + 0.25 Cd
**$BaCd_2Bi_2$**	$P\bar{3}m1$	-21	0.5 $Ba_2Cd_3Bi_4$ + 0.5 Cd
**$BaCd_2N_2$**	$P\bar{3}m1$	371	0.182 $BaCd_{11}$ + 0.182 N_2 + 0.818 BaN_2
**$BaCd_2P_2$**	$P\bar{3}m1$	-135	0.364 BaP_3 + 0.182 $BaCd_{11}$ + 0.455 BaP_2
**$BaCd_2Sb_2$**	$P\bar{3}m1$	-56	0.5 $Ba_2Cd_2Sb_3$ + 0.5 CdSb + 0.5 Cd
**$BaMg_2As_2$**	$P\bar{3}m1$	-178	0.042 Ba_4As_3 + 0.667 Mg_3As_2 + 0.042 $Ba_{20}As_{13}$
**$BaMg_2Bi_2$**	$P\bar{3}m1$	-186	0.545 Mg_3Bi_2 + 0.091 $Ba_{11}Bi_{10}$ + 0.364 Mg
**$BaMg_2N_2$**	*I4/mmm*	51	0.667 Mg_3N_2 + 0.444 Ba_2N + 0.111 BaN_2
**$BaMg_2P_2$**	$P\bar{3}m1$	-163	0.667 Mg_3P_2 + 0.333 Ba_3P_2
**$BaMg_2Sb_2$**	$P\bar{3}m1$	-181	0.6 Mg_3Sb_2 + 0.2 Ba_5Sb_4 + 0.2 Mg
**$BaZn_2As_2$**	*Pnma*	-129	0.326 $Ba_3Zn_2As_4$ + 0.023 $BaZn_{13}$ + 0.349 Zn_3As_2
**$BaZn_2Bi_2$**	*Pnma*	11	0.154 $BaZn_{13}$ + 0.731 Bi + 0.423 Ba_2Bi_3
**$BaZn_2N_2$**	*I4/mmm*	94	0.125 Zn_3N_2 + 0.125 $BaZn_{13}$ + 0.875 BaN_2
**$BaZn_2P_2$**	*Pnma*	-182	0.378 Zn_3P_2 + 0.067 $BaZn_{13}$ + 0.311 Ba_3P_4
**$BaZn_2Sb_2$**	*Pnma*	-70	0.071 $BaZn_{13}$ + 0.929 $BaZnSb_2$ + 0.143 ZnSb
**$CaCd_2As_2$**	$P\bar{3}m1$	-89	0.5 Ca_2CdAs_2 + 0.5 Cd_3As_2
**$CaCd_2Bi_2$**	$P\bar{3}m1$	-22	0.111 $Ca_9Cd_4Bi_9$ + 1 Bi + 1.556 Cd
**$CaCd_2N_2$**	$P\bar{3}m1$	217	2 Cd + 1 CaN_2

**$CaCd_2P_2$**	$P\bar{3}m1$	-105	0.5 Ca_2CdP_2 + 0.5 CdP_2 + 1 Cd
**$CaCd_2Sb_2$**	$P\bar{3}m1$	-61	0.5 Ca_2CdSb_2 + 1 CdSb + 0.5 Cd
**$CaMg_2As_2$**	$P\bar{3}m1$	-159	0.625 Mg_3As_2 + 0.25 Ca_4As_3 + 0.125 Mg
**$CaMg_2Bi_2$**	$P\bar{3}m1$	-148	0.545 Mg_3Bi_2 + 0.091 $Ca_{11}Bi_{10}$ + 0.364 Mg
**$CaMg_2N_2$**	$P\bar{3}m1$	-49	0.667 Mg_3N_2 + 0.333 Ca_3N_2
**$CaMg_2P_2$**	$P\bar{3}m1$	-219	0.5 Mg_3P_2 + 0.5 Mg + 1 CaP
**$CaMg_2Sb_2$**	$P\bar{3}m1$	-135	0.55 Mg_3Sb_2 + 0.1 $Ca_{10}Mg_2Sb_9$ + 0.15 Mg
**$CaZn_2As_2$**	$P\bar{3}m1$	-147	0.002 Ca_4Zn_{51} + 0.047 $Ca_{21}Zn_4As_{18}$ + 0.574 Zn_3As_2
**$CaZn_2Bi_2$**	$P\bar{3}m1$	49	0.039 $Ca4Zn_{51}$ + 0.314 Bi + 0.843 $CaBi_2$
**$CaZn_2N_2$**	$P\bar{3}m1$	-151	0.032 $Ca4Zn_{51}$ + 0.127 Zn_3N_2 + 0.873 CaN_2
**$CaZn_2P_2$**	$P\bar{3}m1$	-185	0.518 Zn_3P_2 + 0.009 Ca_4Zn_{51} + 0.965 CaP
**$CaZn_2Sb_2$**	$P\bar{3}m1$	-47	0.01 Ca_4Zn_{51} + 0.106 $Ca_9Zn_4Sb_9$ + 1.042 ZnSb
**Mg_3As_2**	$Ia\bar{3}$	-332	1 Mg_2As + 1 MgAs
**Mg_3Bi_2**	$Ia\bar{3}$	-187	0.5 Mg_3Bi + 1.5 MgBi
**Mg_3N_2**	$P\bar{3}m1$	-815	1 N_2 + 3 Mg
**Mg_3P_2**	$Ia\bar{3}$	-362	1 Mg_2P + 1 MgP
**Mg_3Sb_2**	$Ia\bar{3}$	-264	1 Mg_2Sb + 1 MgSb
**$MgCd_2As_2$**	$P\bar{3}m1$	56	0.333 Mg_3As_2 + 0.667 Cd_3As_2
**$MgCd_2Bi_2$**	$P\bar{3}m1$	48	0.333 Mg_3Bi_2 + 1.333 Bi + 2 Cd
**$MgCd_2N_2$**	*Pnma*	440	0.667 N_2 + 2 Cd + 0.333 Mg_3N_2
**$MgCd_2P_2$**	$P\bar{3}m1$	59	0.333 Mg_3P_2 + 0.667 CdP_2 + 1.333 Cd
**$MgCd_2Sb_2$**	$P\bar{3}m1$	40	0.333 Mg_3Sb_2 + 1.333 CdSb + 0.667 Cd
**$MgZn_2As_2$**	$P\bar{3}m1$	-7	0.333 Mg_3As_2 + 0.667 Zn_3As_2
**$MgZn_2Bi_2$**	$P\bar{3}m1$	121	0.333 Mg_3Bi_2 + 1.333 Bi + 2 Zn
**$MgZn_2N_2$**	$P\bar{3}m1$	5	0.667 Zn_3N_2 + 0.333 Mg_3N_2
**$MgZn_2P_2$**	$P\bar{3}m1$	-18	0.667 Zn_3P_2 + 0.333 Mg_3P_2
**$MgZn_2Sb_2$**	$P\bar{3}m1$	32	0.333 Mg_3Sb_2 + 1.333 ZnSb + 0.667 Zn
**$SrCd_2As_2$**	$P\bar{3}m1$	-115	0.5 Sr_2CdAs_2 + 0.5 Cd_3As_2
**$SrCd_2Bi_2$**	$P\bar{3}m1$	-24	1 $SrCdBi_2$ + 1 Cd
**$SrCd_2N_2$**	$P\bar{3}m1$	258	0.182 $SrCd_{11}$ + 0.818 SrN_2 + 0.182 N_2
**$SrCd_2P_2$**	$P\bar{3}m1$	-163	0.333 Sr_3P_4 + 0.333 CdP_2 + 1.667 Cd
**$SrCd_2Sb_2$**	$P\bar{3}m1$	-140	0.5 CdSb + 1.5 Cd + 0.5 Sr_2Sb_3
**$SrMg_2As_2$**	$P\bar{3}m1$	-189	0.625 Mg_3As_2 + 0.25 Sr_4As_3 + 0.125 Mg
**$SrMg_2Bi_2$**	$P\bar{3}m1$	-174	0.545 Mg_3Bi_2 + 0.091 $Sr_{11}Bi_{10}$ + 0.364 Mg
**$SrMg_2N_2$**	$P\bar{3}m1$	-32	0.333 Sr_2N + 0.667 Mg_3N_2 + 0.333 SrN
**$SrMg_2P_2$**	$P\bar{3}m1$	-183	0.667 Mg_3P_2 + 0.333 Sr_3P_2
**$SrMg_2Sb_2$**	$P\bar{3}m1$	-190	0.667 Mg_3Sb_2 + 0.02 $Sr_{11}Sb_{10}$ + 0.157 Sr_5Sb_3
**$SrZn_2As_2$**	$P\bar{3}m1$	-83	0.018 $SrZn_{13}$ + 0.491 $Sr_2Zn_2As_3$ + 0.263 Zn_3As_2
**$SrZn_2Bi_2$**	$P\bar{3}m1$	50	0.154 $SrZn_{13}$ + 0.731 Bi + 0.423 Sr_2Bi_3
**$SrZn_2N_2$**	$P\bar{3}m1$	-34	0.154 $SrZn_{13}$ + 0.846 SrN_2 + 0.154 N_2
**$SrZn_2P_2$**	$P\bar{3}m1$	-185	0.034 $SrZn_{13}$ + 0.517 Zn_3P_2 + 0.966 SrP
**$SrZn_2Sb_2$**	$P\bar{3}m1$	-39	0.929 $SrZn_5b_2$ + 0.071 $SrZn_{13}$ + 0.143 ZnSb
**$YbCd_2As_2$**	$P\bar{3}m1$	-169	0.625 Cd_3As_2 + 0.125 Cd + 0.25 Yb_4As_3

**$YbCd_2Bi_2$**	$P\overline{3}m1$	-43	2 Cd + 1 $YbBi_2$
**$YbCd_2N_2$**	$P\overline{3}m1$	183	2 Cd + 1 YbN_2
**$YbCd_2P_2$**	$P\overline{3}m1$	-314	0.333 $YbCd_6$ + 0.333 YbP_5 + 0.333 YbP
**$YbCd_2Sb_2$**	$P\overline{3}m1$	-61	0.5 Yb_2CdSb_2 + 1 CdSb + 0.5 Cd
**$YbMg_2As_2$**	$P\overline{3}m1$	-171	0.625 Mg_3As_2 + 0.125 Mg + 0.25 Yb_4As_3
**$YbMg_2Bi_2$**	$P\overline{3}m1$	-133	0.625 Mg_3Bi_2 + 0.25 Yb_4Bi_3 + 0.125 Mg
**$YbMg_2N_2$**	$P\overline{3}m1$	-82	0.667 Mg_3N_2 + 0.333 Yb_3N_2
**$YbMg_2P_2$**	$P\overline{3}m1$	-371	0.667 Mg_3P_2 + 0.167 Yb_3P + 0.5 YbP
**$YbMg_2Sb_2$**	$P\overline{3}m1$	-148	0.625 Mg_3Sb_2 + 0.25 Yb_4Sb_3 + 0.125 Mg
**$YbZn_2As_2$**	$P\overline{3}m1$	-213	0.01 $YbZn_{11}$ + 0.629 Zn_3As_2 + 0.247 Yb_4As_3
**$YbZn_2Bi_2$**	$P\overline{3}m1$	55	0.182 $YbZn_{11}$ + 0.364 Bi + 0.818 $YbBi_2$
**$YbZn_2N_2$**	$P\overline{3}m1$	-231	0.143 $YbZn_{11}$ + 0.143 Zn_3N_2 + 0.857 YbN_2
**$YbZn_2P_2$**	$P\overline{3}m1$	-387	0.04 $YbZn_{11}$ + 0.52 Zn_3P_2 + 0.96 YbP
**$YbZn_2Sb_2$**	$P\overline{3}m1$	-48	0.478 Yb_2ZnSb_2 + 0.043 $YbZn_{11}$ + 1.043 ZnSb

Table S2: Electronic Structure data for P-3m1 AM_2Pn_2 Compounds

Formula	Indirect Bandgap (eV)	Direct Bandgap (eV)	Bandgap Type (D: Direct, I: Indirect)
**$BaCd_2As_2$**	0.68	0.68	D
**$BaCd_2Bi_2$**	0	0	D
**$BaCd_2N_2$**	0.54	0.57	I
**$BaCd_2P_2$**	1.31	1.31	D
**$BaCd_2Sb_2$**	0.32	0.34	D
**$BaMg_2As_2$**	1.61	1.9	I
**$BaMg_2Bi_2$**	0.49	0.49	D
**$BaMg_2P_2$**	1.75	2.46	I
**$BaMg_2Sb_2$**	1.28	1.86	I
**$BaZn_2As_2$**	0.67	0.77	I
**$BaZn_2Bi_2$**	0	0	D
**$BaZn_2P_2$**	1.1	1.44	I
**$BaZn_2Sb_2$**	0	0	D
**$BaMg_2N_2$**	1.89	2.24	I
**$CaCd_2As_2$**	0.81	0.81	D
**$CaCd_2Bi_2$**	0	0	D
**$CaCd_2N_2$**	0.47	0.54	I
**$CaCd_2P_2$**	1.5	1.5	D
**$CaCd_2Sb_2$**	0.5	0.57	I
**$CaMg_2As_2$**	2	2.2	I
**$CaMg_2Bi_2$**	0.39	0.39	D

**$CaMg_2P_2$**	2.24	3.13	I
**$CaMg_2Sb_2$**	1.37	1.88	I
**$CaZn_2As_2$**	1.02	1.02	D
**$CaZn_2Bi_2$**	0	0	D
**$CaZn_2N_2$**	1.64	1.64	D
**$CaZn_2P_2$**	1.58	1.83	I
**$CaZn_2Sb_2$**	0.12	0.14	D
**$CaMg_2N_2$**	3.03	3.03	D
**$MgCd_2As_2$**	0.4	0.45	I
**$MgCd_2Bi_2$**	0	0	D
**$MgCd_2N_2$**	0.15	0.41	I
**$MgCd_2P_2$**	1.04	1.12	I
**$MgCd_2Sb_2$**	0	0	D
**$MgMn_2Bi_2$**	0	0	D
**$MgMn_2P_2$**	0	0	D
**$MgMn_2Sb_2$**	0.24	1.64	I
**$MgZn_2As_2$**	0.57	1.24	I
**$MgZn_2Bi_2$**	0	0	D
**$MgZn_2N_2$**	1.3	1.3	D
**$MgZn_2P_2$**	1.38	1.94	I
**$MgZn_2Sb_2$**	0	0	D
**Mg_3As_2**	1.49	1.58	I
**Mg_3Bi_2**	0	0	D
**Mg_3N_2**	2.64	2.65	D
**Mg_3P_2**	1.81	2.53	I
**Mg_3Sb_2**	0.55	1.54	I
**$SrCd_2As_2$**	0.71	0.71	D
**$SrCd_2Bi_2$**	0	0	D
**$SrCd_2N_2$**	0.48	0.53	I
**$SrCd_2P_2$**	1.35	1.35	D
**$SrCd_2Sb_2$**	0.34	0.4	I
**$SrMg_2As_2$**	1.94	2.1	I
**$SrMg_2Bi_2$**	0.37	0.37	D
**$SrMg_2P_2$**	2.14	2.86	I
**$SrMg_2Sb_2$**	1.43	1.92	I
**$SrZn_2As_2$**	0.88	0.88	D
**$SrZn_2Bi_2$**	0	0	D
**$SrZn_2N_2$**	1.41	1.41	D
**$SrZn_2P_2$**	1.52	1.66	I
**$SrZn_2Sb_2$**	0.09	0.13	I
**$SrMg_2N_2$**	2.64	2.64	D
**$YbCd_2As_2$**	0.75	0.75	D
**$YbCd_2Bi_2$**	0	0	D

**$YbCd_2N_2$**	0.42	0.51	I
**$YbCd_2P_2$**	1.44	1.44	D
**$YbCd_2Sb_2$**	0.48	0.58	I
**$YbMg_2As_2$**	1.86	2.12	I
**$YbMg_2Bi_2$**	0.33	0.33	D
**$YbMg_2P_2$**	2.09	3.03	I
**$YbMg_2Sb_2$**	1.21	1.84	I
**$YbZn_2As_2$**	1.09	1.1	D
**$YbZn_2Bi_2$**	0	0	D
**$YbZn_2N_2$**	1.65	1.65	D
**$YbZn_2P_2$**	1.45	1.83	I
**$YbZn_2Sb_2$**	0.11	0.15	I
**$YbMg_2N_2$**	2.94	2.94	D

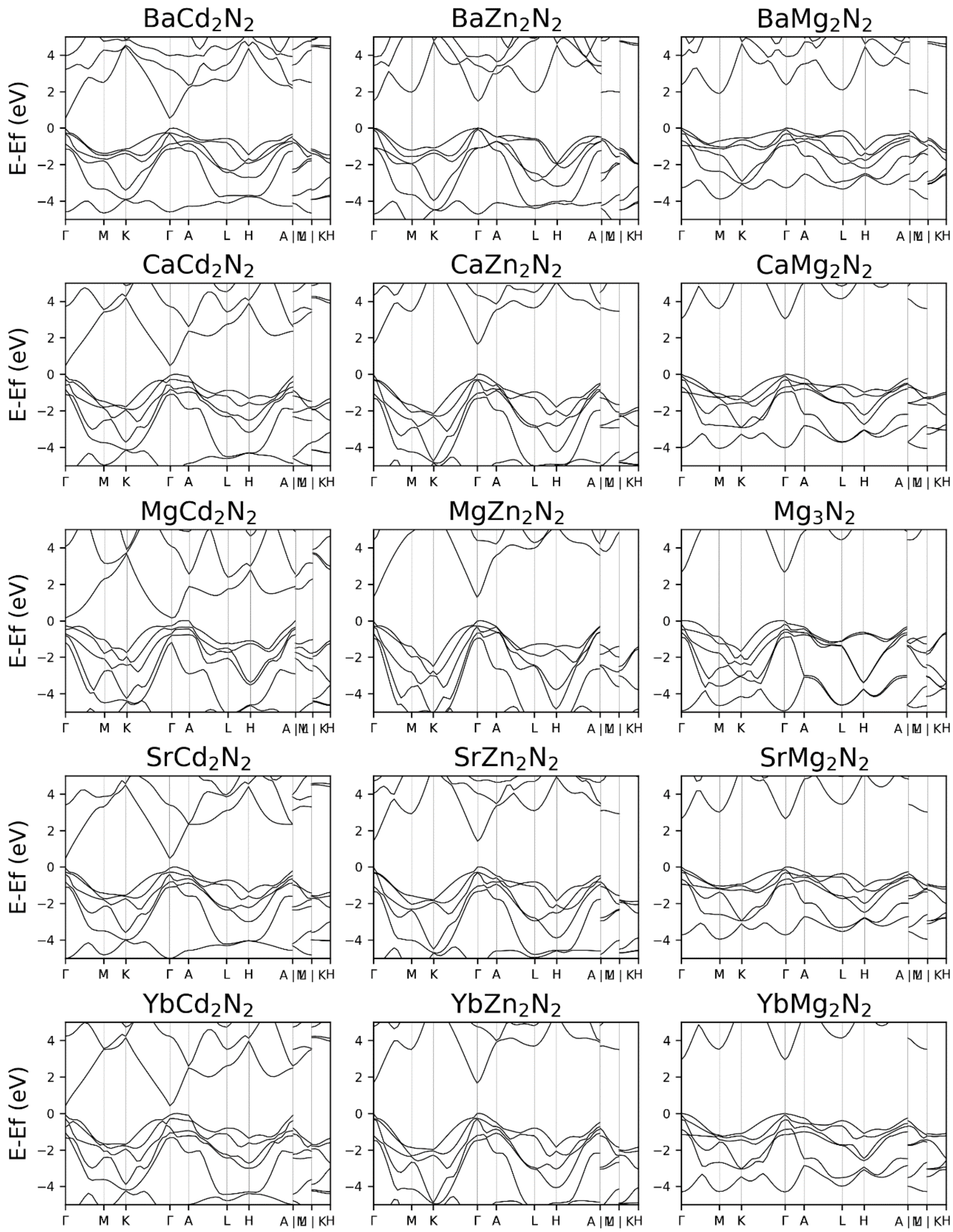
BaCd2N2
BaZn2N2
BaMg2N2
CaCd2N2
CaZn2N2
CaMg2N2
MgCd2N2
MgZn2N2
Mg3N2
SrCd2N2
SrZn2N2
SrMg2N2
YbCd2N2
YbZn2N2
YbMg2N2
E-Ef (eV)
Γ M K Γ A L H A|L M|K H

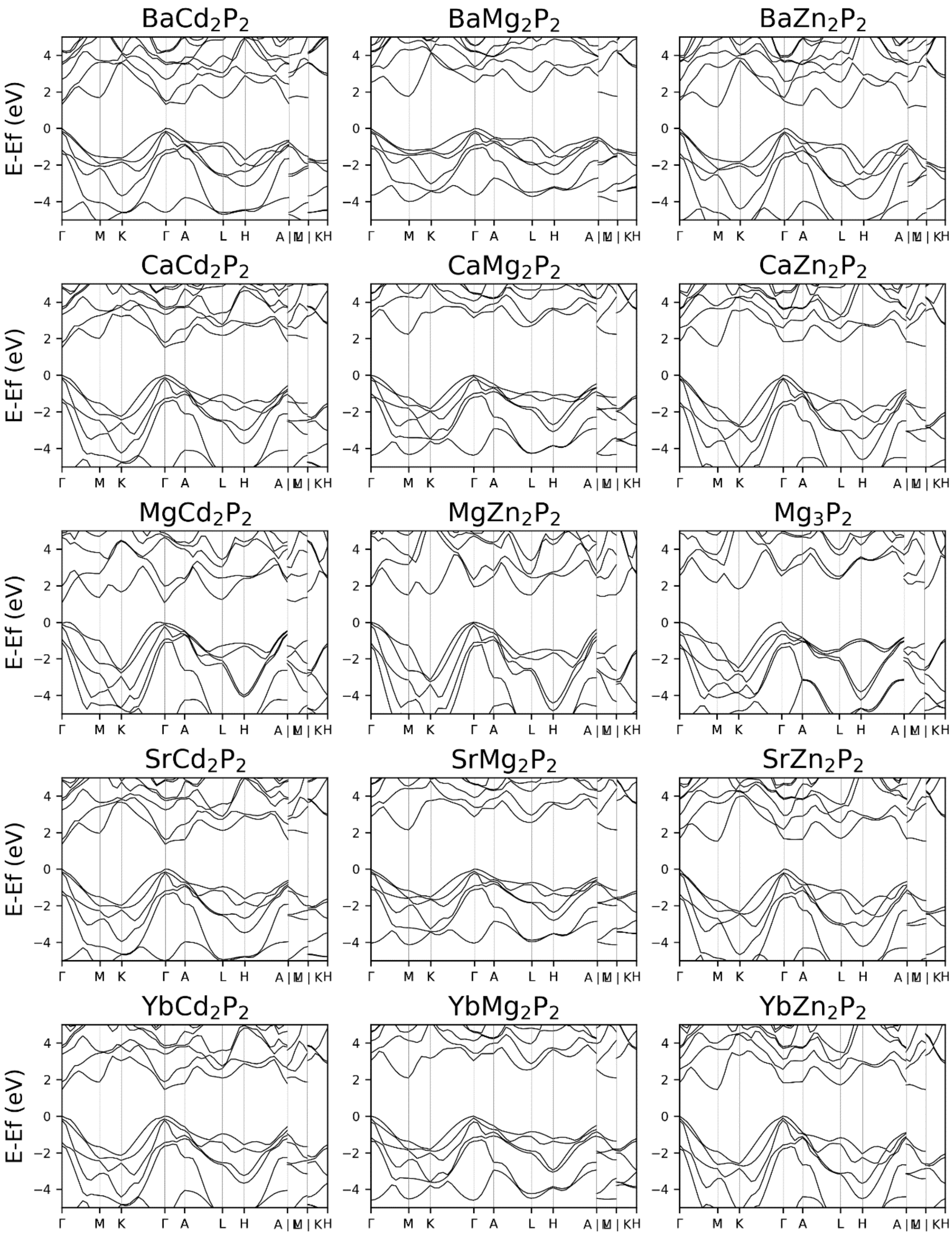
BaCd$_2$P$_2$
BaMg$_2$P$_2$
BaZn$_2$P$_2$
CaCd$_2$P$_2$
CaMg$_2$P$_2$
CaZn$_2$P$_2$
MgCd$_2$P$_2$
MgZn$_2$P$_2$
Mg$_3$P$_2$
SrCd$_2$P$_2$
SrMg$_2$P$_2$
SrZn$_2$P$_2$
YbCd$_2$P$_2$
YbMg$_2$P$_2$
YbZn$_2$P$_2$
E-Ef (eV)
4
2
0
−2
−4
Γ
M
K
Γ
A
L
H
A|L
M|K
H

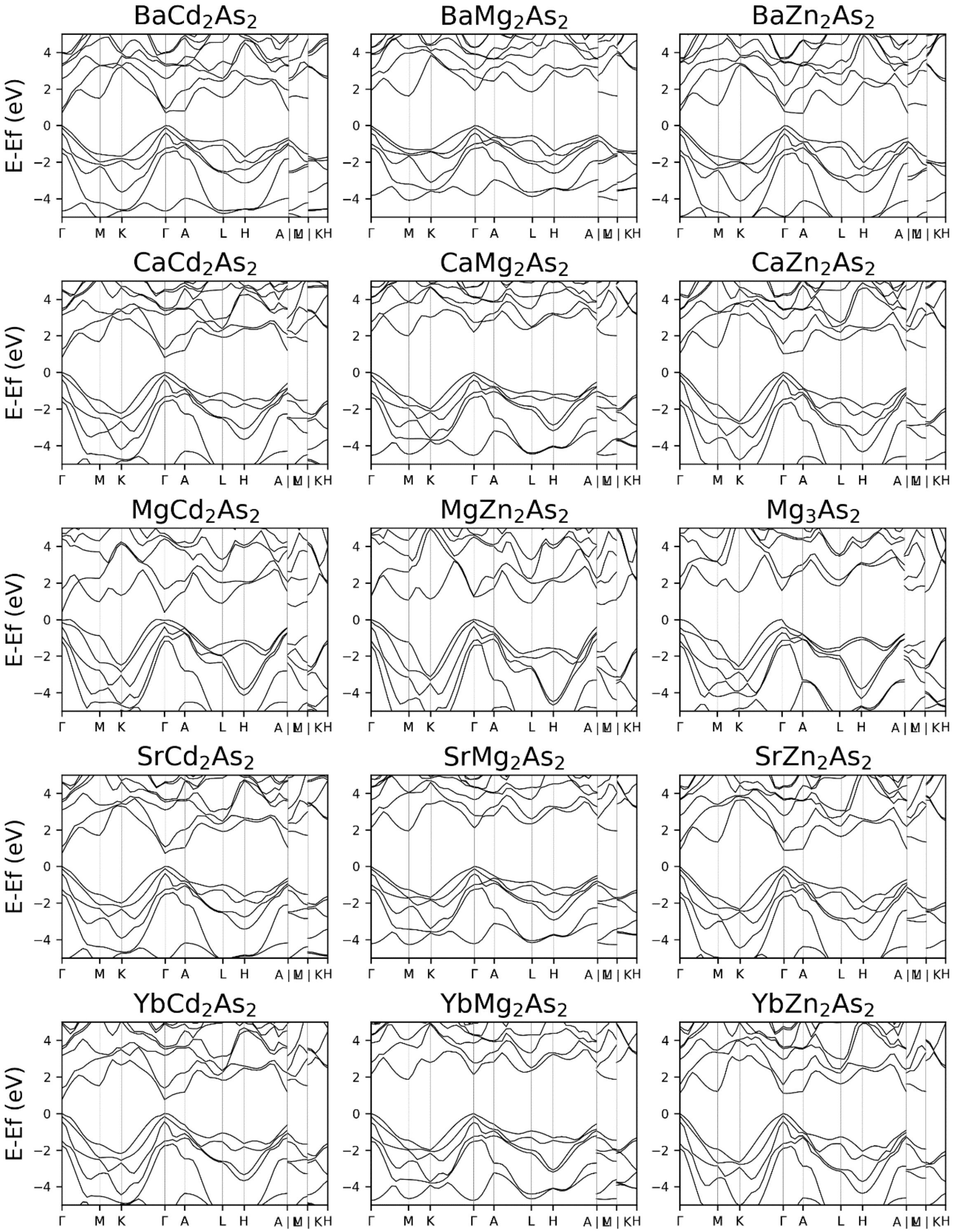
$BaCd_2As_2$
$BaMg_2As_2$
$BaZn_2As_2$
$CaCd_2As_2$
$CaMg_2As_2$
$CaZn_2As_2$
$MgCd_2As_2$
$MgZn_2As_2$
Mg_3As_2
$SrCd_2As_2$
$SrMg_2As_2$
$SrZn_2As_2$
$YbCd_2As_2$
$YbMg_2As_2$
$YbZn_2As_2$
E-Ef (eV)
Γ M K Γ A L H A|L M|K H

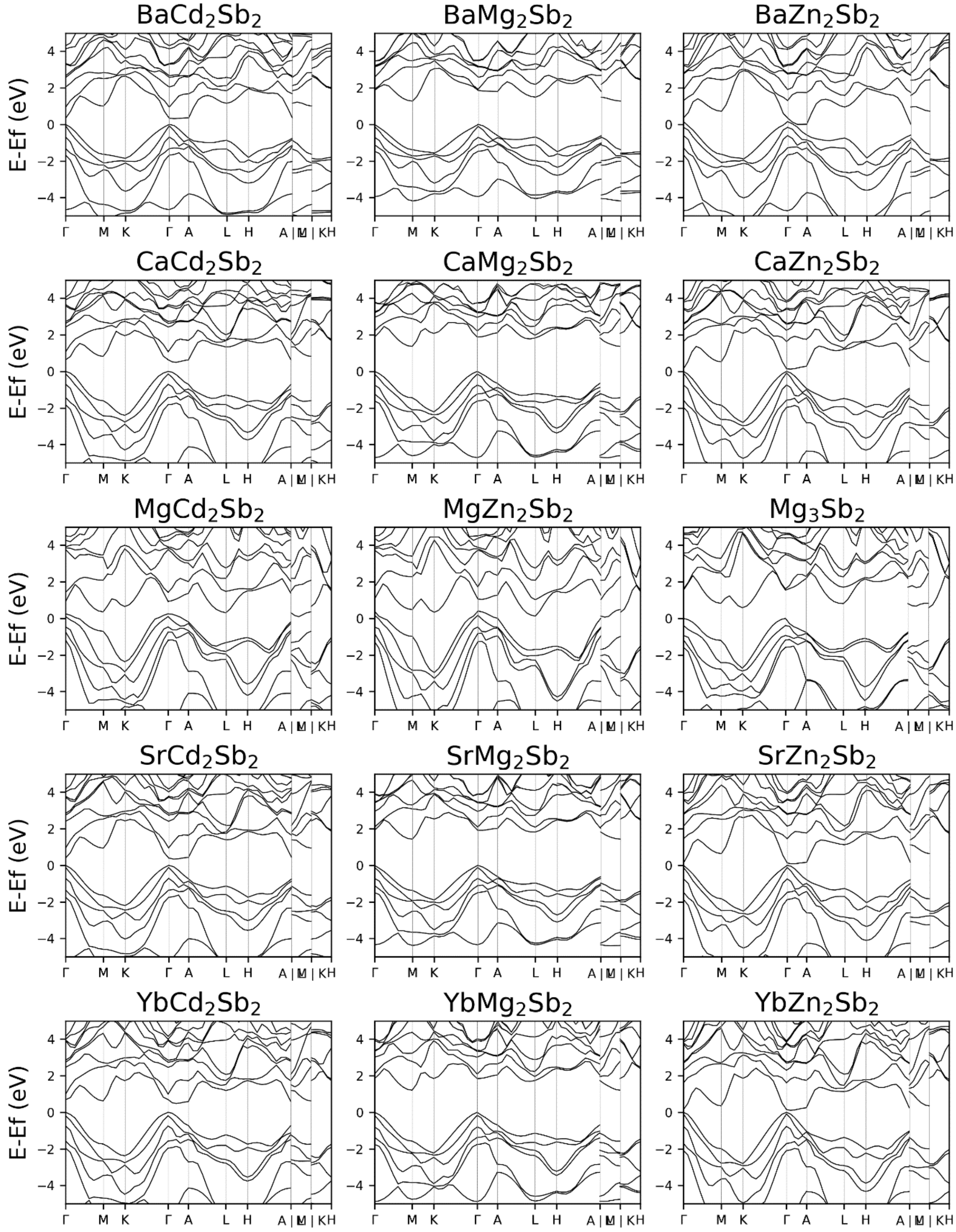
BaCd$_2$Sb$_2$
BaMg$_2$Sb$_2$
BaZn$_2$Sb$_2$
CaCd$_2$Sb$_2$
CaMg$_2$Sb$_2$
CaZn$_2$Sb$_2$
MgCd$_2$Sb$_2$
MgZn$_2$Sb$_2$
Mg$_3$Sb$_2$
SrCd$_2$Sb$_2$
SrMg$_2$Sb$_2$
SrZn$_2$Sb$_2$
YbCd$_2$Sb$_2$
YbMg$_2$Sb$_2$
YbZn$_2$Sb$_2$
E-Ef (eV)
Γ M K Γ A L H A|L M|K H

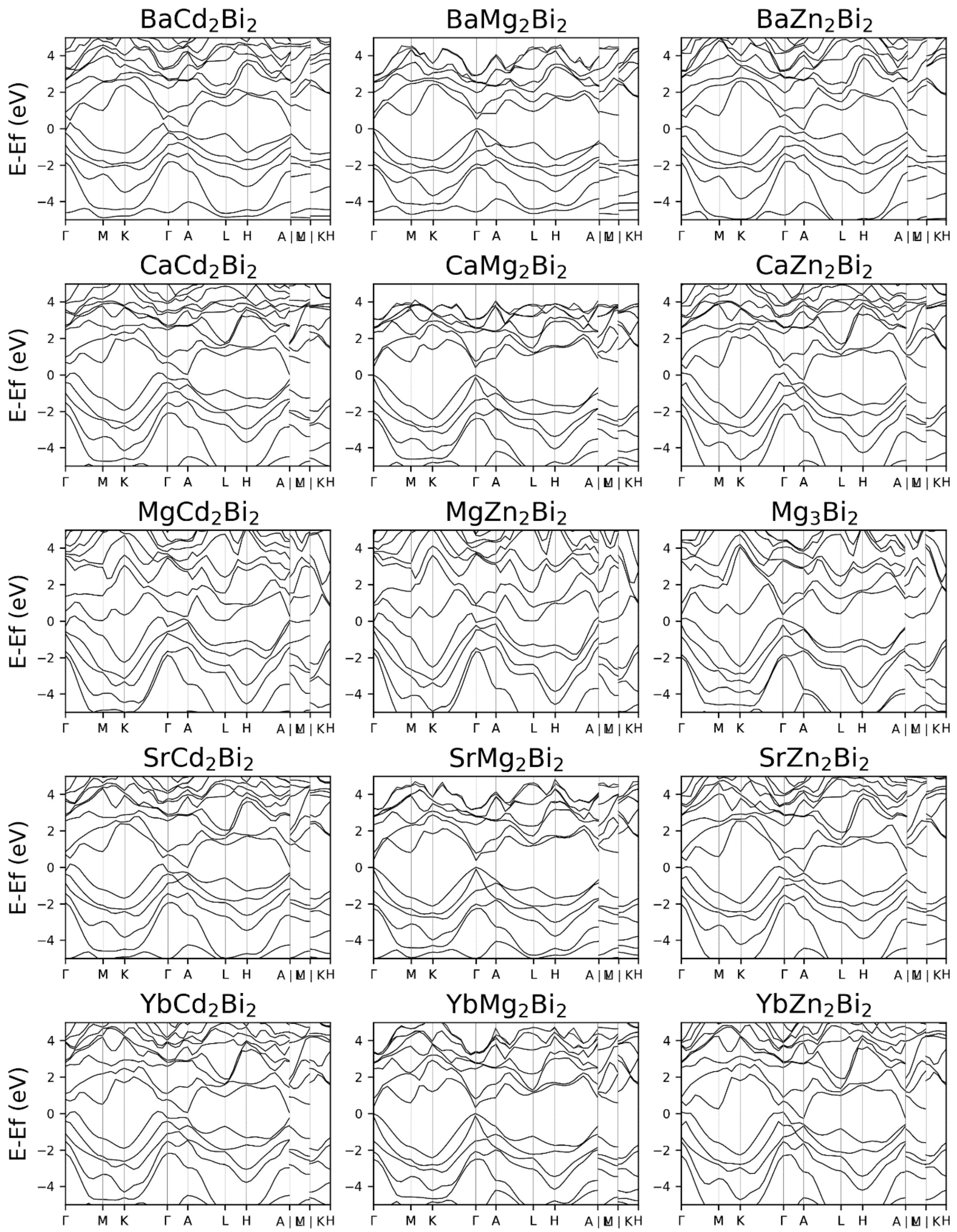

Fig. S4 – HSE+SOC band structures of AM_2Pn_2 compounds in the $P\bar{3}m1$ space group structure.

SI Section 2: Comparison of Experimentally Reported Bandgaps and HSE+SOC on PBE Calculated Bandgaps

We find several discrepancies between our calculated bandgaps and those reported in the literature that we have chosen to exclude from the MAE of our comparison, which we will discuss now. First, several materials have their bandgaps reported several times independently, so we can compare between those. Ponnambalam et. al [2] report a bandgap of 0.6 eV for $CaZn_2P_2$ deduced from temperature dependence of electrical resistivity in powder/crystal samples, whereas our calculations show a much larger indirect bandgap of 1.58 eV. Further experimental reports also claim a higher bandgap in agreement with our calculations; 1.85 eV in Katsube et al. [3] by diffuse reflectance and 1.6 eV in Quadir et al. [4] by photoluminescence spectroscopy, leading us to conclude Ponnambalam et al. [2] misrepresented the bandgap of $CaZn_2P_2$. They also report the bandgap of YbZn2P2 to be 0.4 eV in the same publication, whereas our calculations show an indirect bandgap of 1.45 eV. To the best of our knowledge there are no other experimental reports for the bandgap of this material, but given the previous inaccuracy of their $CaZn_2P_2$ bandgap measurement, we believe this report is likely incorrect as well. Since the bandgaps of this class of materials are not very well explored, repeated bandgap measurements are somewhat sparse, so next we look at compounds that have only one reported bandgap measurement, but have similar compounds with reported bandgap measurements. Chen et al. [5] report the bandgap of $SrCd_2As_2$ is 0.21 eV via the change in electrical resistivity with respect to temperature, whereas we have calculated it to be 0.71 eV (nearly direct). While there do not seem to be other reports of the bandgap of $SrCd_2As_2$, $BaCd_2As_2$ has been reported to have a bandgap of 0.4 eV with and 0.84 eV by Kunioka et al. [6] and Yang et al. [7] , respectively, both also via electrical resistivity, and $SrCd_2Sb_2$ has been reported as 0.63 by Jin et al. [8] from a Tauc plot of optical absorbance. Based off the trend in atomic mass, $SrCd_2As_2$ should have a bandgap higher than $BaCd_2As_2$ or $SrCd_2Sb_2$ since it is lighter, corroborating our calculations. Similarly, Wang et al.'s [9] 0.7 eV measurement of the bandgap of $BaCd_2Sb_2$ does not match with our calculations (0.32 eV, direct) or reports of similar compounds: $BaCd_2As_2$'s bandgap of 0.4 and 0.84 eV by Kunioka et al. [6] and Yang et al. [7] and $SrCd_2Sb_2$'s bandgap of 0.63 eV measured by Jin et al. [8]. So, we believe 0.7 eV would be too high to realistically be the $BaCd_2Sb_2$'s bandgap since it should be lower than $BaCd_2As_2$ and $SrCd_2Sb_2$. For $CaZn_2N_2$ it appears our calculations estimate the bandgap to be too low, 1.64 eV nearly direct, versus experimental reports of 1.9 and 1.93 eV from Hinuma et al. [10] and Tsuji et al. [11] respectively. HSE band structure calculations based off of a prior HSE relaxation show a bandgap of 1.90, which matches the experimental value very well.

The reported band gaps for $CaMg_2N_2$ in the literature are quite scattered. Based on Tauc plot method, Ma et Al. [12] reported a band gap of 2.56 eV, while Hinuma et Al. [10] found a band gap of 3.25 eV. Our HSE06 calculated bandgap is direct and has an energy of 3.0 eV. Given the underestimation noted for our calculations for $CaZn_2N_2$, it is likely our HSE06+PBE calculation for the bandgap of $CaMg_2N_2$ is underestimated as well, so we do an HSE relaxation and HSE+SOC band structure calculation. This more advanced calculation gives a bandgap of 3.25 eV, matching the results of Hinuma et al. [10].As another check, the HSE+SOC band structure calculation on the PBEsol relaxed structure gives a bandgap of 3.26 eV. So, noting a similar error for $CaZn_2N_2$ it seems that our data has a slight underestimation of the bandgaps for some nitrides due to

overestimation of the lattice constants by PBE, where HSE would have been better for the relaxation, or PBEsol at a similar computational cost to PBE. The only other reported nitride bandgaps in this class are $SrZn_2N_2$ by Kikuchi et al. [13] (error of 0.2 eV) and Mg_3N_2 [14] by Fang et al. and Ma et al. [12] (error of 0.15 and -0.06 eV, respectively), which agree decently well. Though without more reports of the bandgaps of AM_2N_2 compounds it is difficult to say how pervasive this trend is.

Table S3 - Literature comparison for bandgaps of AM_2Pn_2 compounds

Formula	Bandgap (HSE+SOC minimum)	Bandgap (experimentally reported)	Difference	Measurement Method	Citation
**$BaCd_2As_2$**	0.68	0.4	-0.28	Thermal - resistivity	6
**$BaCd_2As_2$**	0.68	0.84	-0.26	Thermal – resistivity	7
**$BaCd_2P_2$**	1.31	1.46	0.15	Optical – PL spectroscopy	15
**$BaCd_2Sb_2$**	0.32	0.7	0.38	Optical – Tauc plot	9
**$CaMg_2Bi_2$**	0.40	0.2	-0.19	Optical – Tauc plot	16
**$CaMg_2N_2$**	3.03	2.56	-0.47	Optical – Tauc plot	12
**$CaZn_2N_2$**	1.64	1.93	0.29	Optical – Tauc plot	11
**$CaZn_2N_2$**	1.64	1.9	0.26	Optical - diffuse reflectance	10
**$CaZn_2P_2$**	1.58	0.6	-0.98	Thermal – resistivity	2
**$CaZn_2P_2$**	1.58	1.85	0.27	Optical – PL spectroscopy	3
**$CaZn_2P_2$**	1.58	1.6	0.02	diffuse reflectance	4
**$CaZn_2Sb_2$**	0.12	0.26	0.14	Thermal – resistivity	17
**$CaZn_2Sb_2$**	0.12	0.26	0.14	Thermal – resistivity	18
**Mg_3Bi_2**	0	0	0	Thermal - Goldsmid Sharp	19
**Mg_3N_2**	2.64	2.8	0.16	Optical - diffuse reflectance	14
**Mg_3N_2**	2.64	2.58	-0.06	Optical – Tauc plot	12
**Mg_3Sb_2**	0.55	0.54	-0.01	Thermal -Goldsmid Sharp	19
**$SrCd_2As_2$**	0.71	0.21	-0.50	Thermal – resistivity	5
**$SrCd_2Sb_2$**	0.34	0.63	0.29	Optical – Tauc plot	8
**$SrZn_2N_2$**	1.41	1.6	0.19	Optical – Tauc plot	13
**$SrZn_2P_2$**	1.52	1.7	0.18	diffuse reflectance	3
**$SrZn_2Sb_2$**	0.09	0.27	0.18	Thermal – resistivity	17
**$YbCd_2Sb_2$**	0.48	0.55	0.07	Optical – Tauc plot	20
**$YbMg_2Bi_2$**	0.33	0.3	-0.03	Thermal – resistivity	16
**$YbZn_2P_2$**	1.45	0.4	-1.05	Thermal – resistivity	2
**$YbZn_2Sb_2$**	0.11	0	-0.11	Thermal - resistivity	17
**$YbZn_2Sb_2$**	0.11	0.55	0.44	Optical – Tauc plot	20
**$YbZn_2Sb_2$**	0.11	0	-0.11	Not listed	18

A)

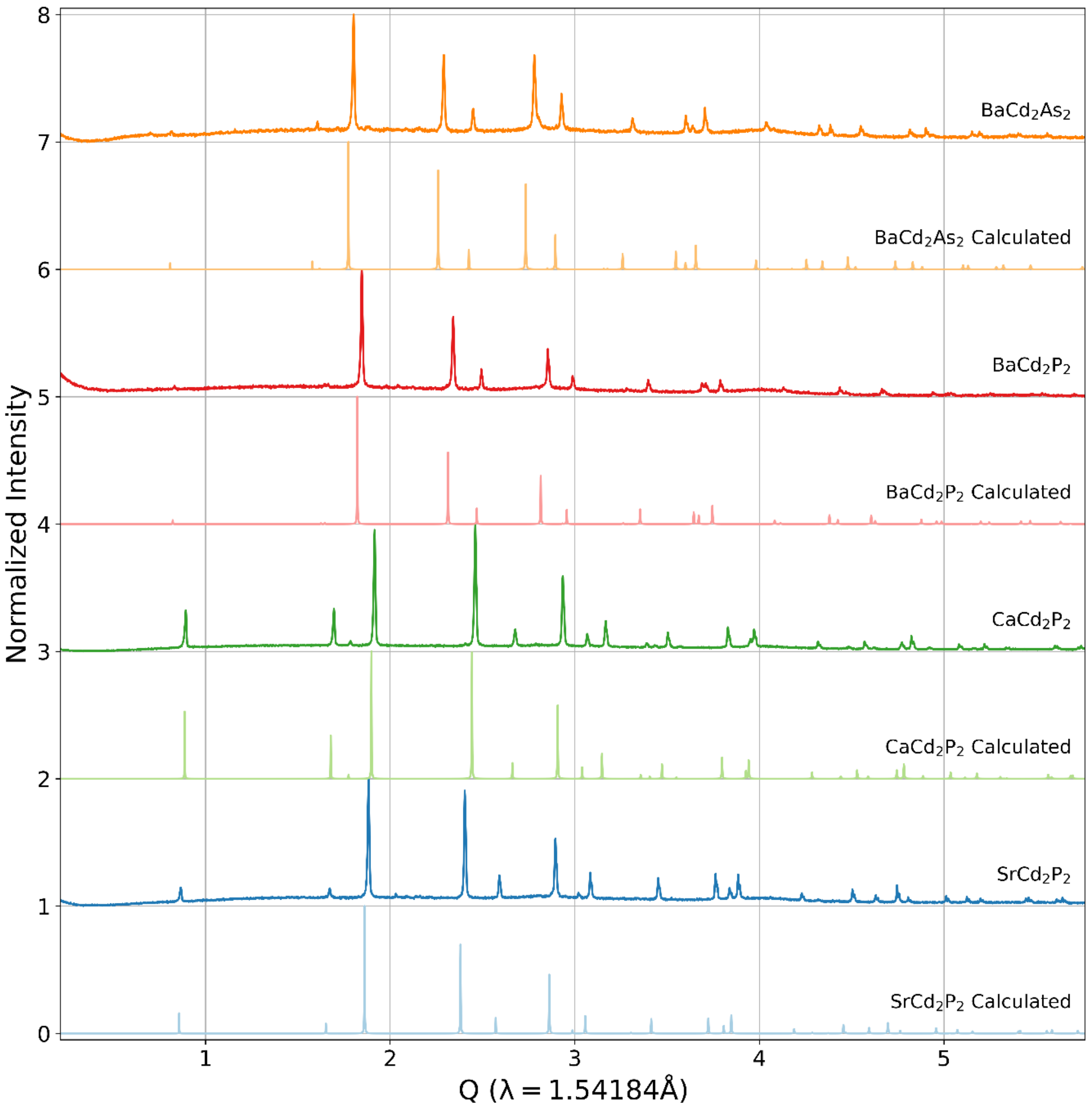

BaCd2As2
BaCd2As2 Calculated
BaCd2P2
BaCd2P2 Calculated
CaCd2P2
CaCd2P2 Calculated
SrCd2P2
SrCd2P2 Calculated
Normalized Intensity
Q (λ = 1.54184Å)

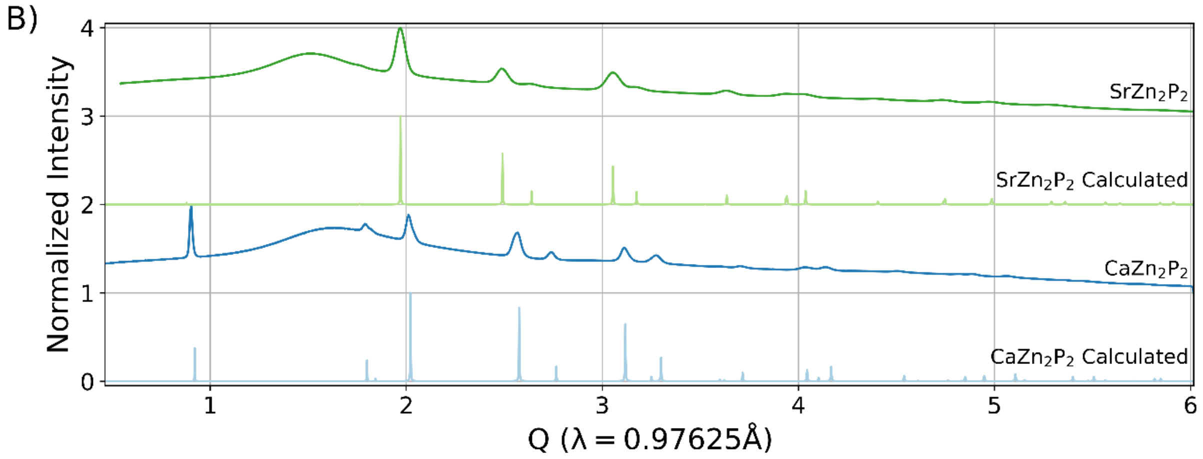

Fig. S5 – X-ray diffraction patterns (XRD) of synthesized phases in A) powder B) thin films. Calculated patterns were generated in VESTA[21] from PBE-relaxed $P\bar{3}m1$ unit cells. Note the amorphous background in the thin film samples is attributable to the amorphous silica substrate.

Table S4 – Comparison of experimental and PBE lattice parameters

Compound	Lattice Parameter (Å, Experiment)	Lattice Parameter (Å, PBE)	Sample type	Error (%)
$BaCd_2As_2$	a=4.5153(1)<br>c=7.6909(1)	a= 4.607<br>c= 7.787	Powder	a : 2.03<br>c: 1.25
$BaCd_2P_2$	a=4.3997(1)<br>c=7.5531(2)	a= 4.462<br>c= 7.633	Powder	a: 1.42<br>c: 1.06
$CaCd_2P_2$	a=4.2741(4)<br>c=7.0258(6)	a= 4.317<br>c= 7.060	Powder	a: 1.00<br>c: 0.50
$SrCd_2P_2$	a=4.3376(1)<br>c=7.2671(2)	a= 4.394<br>c= 7.342	Powder	a: 1.30<br>c: 1.03
$SrZn_2P_2$	a = 4.1172<br>c = 7.1506	a= 4.1021<br>c= 7.1049	Thin film	a: -0.37<br>c: -0.64
$CaZn_2P_2$	a = 4.0333<br>c = 6.898	a= 4.0233<br>c= 6.791	Thin film	a: -0.24<br>c: -1.55

Table S5 – Pseudopotential versions

Element	VASP pseudopotential name
Ba	Ba_sv
Sr	Sr_sv
Ca	Ca_sv
Yb	Yb_2
Mg	Mg_pv

Cd	Cd
Zn	Zn
N	N
P	P
As	As
Sb	Sb
Bi	Bi

Table S6 - Lattice parameters of Mg_3Sb_2

Space group Symbol	Experimental	PBE	PBEsol	R2SCAN
P-3m1	a=4.568 [22]<br>c=7.229	a=4.602<br>c=7.255	a=4.561<br>c=7.215	a=4.571<br>c=7.253
Ia-3	a=13.48(2) [23]	a=13.434	a=13.298	a=13.326

Table S7 - Energy Difference of $P\overline{3}m1$ and $Ia\overline{3}$ Mg_3Sb_2

Functional	Energy difference (meV/atom)
PBE	16
PBEsol	-42
r2SCAN	28

(1) Klüfers, P.; Mewis, A. AB2X2- Verbingdungen Mit CaAl2Si2-Struktur X. Zur Struktur Neuer Ternärer Erdalkaliphosphide Und -Arsenide. *Z Kristallogr Cryst Mater* **1984**, *169* (1–4), 135–148. https://doi.org/doi:10.1524/zkri.1984.169.14.135.

(2) Ponnambalam, V.; Lindsey, S.; Xie, W.; Thompson, D.; Drymiotis, F.; Tritt, T. M. High Seebeck Coefficient AMXP 2 (A = Ca and Yb; M, X = Zn, Cu and Mn) Zintl Phosphides as High-Temperature Thermoelectric Materials. *J Phys D Appl Phys* **2011**, *44* (15), 155406. https://doi.org/10.1088/0022-3727/44/15/155406.

(3) Katsube, R.; Nose, Y. Synthesis of Alkaline-Earth Zintl Phosphides MZn2P2 (M = Ca, Sr, Ba) from Sn Solutions. *High Temperature Materials and Processes* **2022**, *41* (1), 8–15. https://doi.org/10.1515/htmp-2022-0019.

(4) Quadir, S.; Yuan, Z.; Esparza, G. L.; Dugu, S.; Mangum, J. S.; Pike, A.; Hasan, M. R.; Kassa, G.; Wang, X.; Coban, Y.; Liu, J.; Kovnir, K.; Fenning, D. P.; Reid, O. G.; Zakutayev, A.; Hautier, G.; Bauers, S. R. Low-Temperature Synthesis of Stable CaZn2P2 Zintl Phosphide Thin Films as Candidate Top Absorbers. *Adv Energy Mater* **2024**, *14* (44), 2402640. https://doi.org/10.1002/aenm.202402640.

(5) Chen, B.; Deng, Z.; Li, W.; Gao, M.; Li, Z.; Zhao, G.; Yu, S.; Wang, X.; Liu, Q.; Jin, C. (Sr1- x Na x)(Cd1- x Mn x)2As2: A New Charge and Spin Doping Decoupled Diluted Magnetic Semiconductors with CaAl2Si2-Type Structure. *J Appl Phys* **2016**, *120* (8), 083902. https://doi.org/10.1063/1.4961565.

(6) Kunioka, H.; Kihou, K.; Nishiate, H.; Yamamoto, A.; Usui, H.; Kuroki, K.; Lee, C. H. Thermoelectric Properties of (Ba,K)Cd2As2 Crystallized in the CaAl2Si2-Type Structure. *Dalton Transactions* **2018**, *47* (45), 16205–16210. https://doi.org/10.1039/c8dt02955e.

(7) Yang, X.; Li, Y.; Zhang, P.; Jiang, H.; Luo, Y.; Chen, Q.; Feng, C.; Cao, C.; Dai, J.; Tao, Q.; Cao, G.; Xu, Z.-A. K and Mn Co-Doped BaCd2As2: A Hexagonal Structured Bulk Diluted Magnetic Semiconductor with Large Magnetoresistance. *J Appl Phys* **2013**, *114* (22), 223905. https://doi.org/10.1063/1.4842875.

(8) Jin, M.; Zheng, L.; Sun, C.; Jiang, L.; Meng, X.; Chen, Q.; Li, W. Manipulation of Hole and Band for Thermoelectric Enhancements in SrCd2Sb2 Zintl Compound. *Chemical Engineering Journal* **2021**, *420*, 130530. https://doi.org/10.1016/j.cej.2021.130530.

(9) Wang, X.; Li, W.; Zhou, B.; Sun, C.; Zheng, L.; Tang, J.; Shi, X.; Pei, Y. Experimental Revelation of Multiband Transport in Heavily Doped BaCd2Sb2 with Promising Thermoelectric Performance. *Materials Today Physics* **2019**, *8*, 123–127. https://doi.org/10.1016/j.mtphys.2019.03.002.

(10) Hinuma, Y.; Hatakeyama, T.; Kumagai, Y.; Burton, L. A.; Sato, H.; Muraba, Y.; Iimura, S.; Hiramatsu, H.; Tanaka, I.; Hosono, H.; Oba, F. Discovery of Earth-Abundant Nitride Semiconductors by Computational Screening and High-Pressure Synthesis. *Nat Commun* **2016**, *7* (1), 11962. https://doi.org/10.1038/ncomms11962.

(11) Tsuji, M.; Hanzawa, K.; Kinjo, H.; Hiramatsu, H.; Hosono, H. Heteroepitaxial Thin-Film Growth of a Ternary Nitride Semiconductor CaZn2N2. *ACS Appl Electron Mater* **2019**, *1* (8), 1433–1438. https://doi.org/10.1021/acsaelm.9b00248.

(12) Ma, B.; Ding, J.; Long, Q.; Wang, Y. The Mechanism of N-Vacancy Defects Self-Activated Light Emitting Based on CaMg2N2. *J Lumin* **2019**, *208* (August 2018), 388–393. https://doi.org/10.1016/j.jlumin.2019.01.002.

(13) Kikuchi, R.; Ueno, K.; Nakamura, T.; Kurabuchi, T.; Kaneko, Y.; Kumagai, Y.; Oba, F. SrZn2N2as a Solar Absorber: Theoretical Defect Chemistry and Synthesis by Metal Alloy Nitridation. *Chemistry of Materials* **2021**, *33* (8), 2864–2870. https://doi.org/10.1021/acs.chemmater.1c00075.

(14) Fang, C. M.; De Groot, R. A.; Bruls, R. J.; Hintzen, H. T.; De With, G. Ab Initio Band Structure Calculations of Mg3N2 and MgSiN2. *Journal of Physics Condensed Matter* **1999**, *11* (25), 4833–4842. https://doi.org/10.1088/0953-8984/11/25/304.

(15) Yuan, Z.; Dahliah, D.; Hasan, M. R.; Kassa, G.; Pike, A.; Quadir, S.; Claes, R.; Chandler, C.; Xiong, Y.; Kyveryga, V.; Yox, P.; Rignanese, G. M.; Dabo, I.; Zakutayev, A.; Fenning, D. P.; Reid, O. G.; Bauers, S.; Liu, J.; Kovnir, K.; Hautier, G. Discovery of the Zintl-Phosphide BaCd2P2 as a Long Carrier Lifetime and Stable Solar Absorber. *Joule* **2024**, *8* (5), 1412–1429. https://doi.org/10.1016/j.joule.2024.02.017.

(16) May, A. F.; McGuire, M. A.; Singh, D. J.; Ma, J.; Delaire, O.; Huq, A.; Cai, W.; Wang, H. Thermoelectric Transport Properties of CaMg2Bi2, EuMg2Bi2, and YbMg2Bi2. *Phys Rev B* **2012**, *85* (3), 035202. https://doi.org/10.1103/PhysRevB.85.035202.

(17) Christensen, M.; Johnsen, S.; Iversen, B. B. Thermoelectric Clathrates of Type i. *Dalton Transactions* **2010**, *39* (4), 978–992. https://doi.org/10.1039/b916400f.

(18) Guo, K.; Cao, Q.; Zhao, J. Zintl Phase Compounds AM2Sb2 (A=Ca, Sr, Ba, Eu, Yb; M=Zn, Cd) and Their Substitution Variants: A Class of Potential Thermoelectric Materials. *Journal of Rare Earths* **2013**, *31* (11), 1029–1038. https://doi.org/10.1016/S1002-0721(12)60398-6.

(19) Imasato, K.; Kang, S. D.; Ohno, S.; Snyder, G. J. Band Engineering in Mg3Sb2 by Alloying with Mg3Bi2 for Enhanced Thermoelectric Performance. *Mater Horiz* **2018**, *5* (1), 59–64. https://doi.org/10.1039/c7mh00865a.

(20) Wang, X.; Li, J.; Wang, C.; Zhou, B.; Zheng, L.; Gao, B.; Chen, Y.; Pei, Y. Orbital Alignment for High Performance Thermoelectric YbCd2Sb2 Alloys. *Chemistry of Materials* **2018**, *30* (15), 5339–5345. https://doi.org/10.1021/acs.chemmater.8b02155.

(21) Momma, K.; Izumi, F. VESTA3 for Three-Dimensional Visualization of Crystal, Volumetric and Morphology Data. *J Appl Crystallogr* **2011**, *44* (6), 1272–1276. https://doi.org/10.1107/S0021889811038970.

(22) Martinez-Ripoll, M.; Haase, A.; Brauer, G. The Crystal Structure of α-Mg 3 Sb 2. *Acta Crystallogr B* **1974**, *30* (8), 2006–2009. https://doi.org/10.1107/S0567740874006285.

(23) Sevast’yanova, L. G.; Kravchenko, O. V.; Gulish, O. K.; Stupnikov, V. A.; Leonova, M. E.; Zhizhin, M. G. Binary and Ternary Compounds in the Mg-Sb-B and Mg-Bi-B Systems as Catalysts for the Synthesis of Cubic BN. *Inorganic Materials* **2006**, *42* (8), 863–866. https://doi.org/10.1134/S0020168506080115.
